# Influence of Beeswax Content on Barrier Properties of PVA-Based Films

**DOI:** 10.3390/foods15214006

**Published:** 2026-11-09

**Authors:** Lucica Maria Pop, Camelia Nicula, Alexandra Maria Uivarasan, Zorica Voșgan, Monica Liliana Marian, Cristina Mihali, Lucia Mihalescu, Oana Mare Rosca, Monica Tarau, Ionela Traistaru Marcu, Adrian Matei Cosma, Doina Boitor Hontau, Goran Drazic, Klara Magyari, Marieta Muresan-Pop, Ioan Botiz, Milica Todea, Lucian Baia, Anca Peter

**Affiliations:** 1 Faculty of Sciences, Technical University of Cluj Napoca, Victoriei 76, 430072 Baia Mare, Romania; lucica.pop@campus.utcluj.ro (L.M.P.); camelia.nicula@cb.utcluj.ro (C.N.); alexandra.uivarasan@cb.utcluj.ro (A.M.U.); zorica.vosgan@cb.utcluj.ro (Z.V.); monica.marian@cb.utcluj.ro (M.L.M.); cristina.mihali@cb.utcluj.ro (C.M.); lucia.mihalescu@cb.utcluj.ro (L.M.); oana.mare@cb.utcluj.ro (O.M.R.); monica.tarau@campus.utcluj.ro (M.T.); ionela.traistaru@campus.utcluj.ro (I.T.M.); adrian.cosma@campus.utcluj.ro (A.M.C.); doina.hontau@campus.utcluj.ro (D.B.H.); 2 National Institute of Chemistry, Hajdrihova 19, P.O. Box 660, SI-1001 Ljubljana, Slovenia; goran.drazic@gmail.com; 3 Interdisciplinary Research Institute on Bio-Nano-Sciences, Babes-Bolyai University, Nanostructured Materials and Bio-Nano-Interface Center, 42, Treboniu Laurian, 400271 Cluj-Napoca, Romania; klara.magyari@ubbcluj.ro (K.M.); marieta.muresan@ubbcluj.ro (M.M.-P.); ioan.botiz@ubbcluj.ro (I.B.); militodea@yahoo.com (M.T.); lucian.baia@ubbcluj.ro (L.B.); 4 Faculty of Physics, Babes-Bolyai University, M. Kogălniceanu 1, 400084 Cluj-Napoca, Romania; 5 Institute for Research-Development-Innovation in Applied Natural Sciences, Babes-Bolyai University, Fântânele 30, 400327 Cluj-Napoca, Romania; 6 Faculty of Nursing and Health Sciences, Iuliu Hațieganu University of Medicine and Pharmacy, Victor Babeș 8, 400012 Cluj-Napoca, Romania

**Keywords:** elemental analysis, thermogravimetrical properties, water vapor transmission rate, solubility in food simulants, UV-blocking performance, tensile strength

## Abstract

The aim of this study was to establish the optimal content of beeswax (BW) that can be added to a polyvinyl alcohol (PVA) matrix in order to obtain the maximum barrier effect against water vapor. The beeswax, ranging from 0 wt.% to 17.79 wt.%, induced surface heterogeneity and increased roughness. Although migration in all the studied simulants exceeded the limit of 10 mg/dm^2^/24 h, of all the prepared films, the most efficient ones were found to be those containing 12.63–15.5 wt.% beeswax, which exhibited 50% lower solubility in aqueous, acidic, alcoholic, and fatty food simulants than did neat PVA. The disadvantage is that the tensile strength of the films is 16 times less than PE and 8 times less than unchanged PVA when 12.63–17.79 wt.% BW is added. The water vapor transmission rate was 24 times lower than that of neat PVA and only 2 times higher than that of polyethylene (PE). Additionally, the results confirmed the separation of PVA and beeswax phases with the addition of more than 15.5 wt.% beeswax. BW incorporation modifies the high-temperature degradation pathway of PVA, leading to more substantial mass losses, but without compromising the overall thermal stability of the films. This is a preliminary study that established that the maximum hydrophobic effect is obtained at a beeswax content of 15.29 wt%. The preparation recipe must be optimized by testing with other hydrophobic agents that reduce migration in simulants and increase the barrier properties, and nanoparticles that increase mechanical strength.

## 1. Introduction

Biodegradable polymers may be a viable substitute for traditional non-biodegradable food packaging materials due to societal environmental concerns [1]. As a result, there has been a growing interest in biodegradable polymers, whether they are derived from biomass or renewable sources, plant or animal, or biodegradable synthetic polymers like polyvinyl alcohol (PVA), polyhydroxybutyrate (PHB), or polylactic acid (PLA) [2]. It is crucial that these polymers have suitable properties that allow them to withstand physical stress, as well as stable thermal properties, in order to preserve their structural integrity under the conditions of technological and storage processes, and effective barrier properties against gases, water vapor, and volatile substances to preserve the freshness and quality of food. Further research and development are required to make these materials suitable for food packaging applications because they typically have lower barrier performance than traditional plastics [3].

PVA is a synthetic, biodegradable, semicrystalline, non-toxic, and biocompatible polymer. It has good film-forming qualities and is widely used in pharmaceutical and medical industries [4], as well as in the food industry as food packaging material. PVA is a biopolymer that is insoluble in organic solvents but dissolves in water. It has low light and oxygen permeability, excellent mechanical properties, and high thermal stability. However, due to its high permeability to water vapor and the presence of highly polar hydroxyl groups in its structure, its hydrophilic nature limits its use in packaging applications [5,6]. A packaging material’s hydrophilic nature can offer a number of benefits, such as superior film-forming ability, gradual bioactive compound diffusion, improved adhesion, etc. [7]. However, there are some drawbacks to its widespread use, such as the fact that it offers insufficient protection against water vapor, which can result in the growth of microorganisms and the loss of food safety and quality, ultimately leading to food degradation [7]. Researchers have been diligently working to develop solutions for hydrophobic packaging films to increase the potential of PVA-based packaging materials in environments that require moisture resistance and to reduce food waste. By forming an efficient barrier against water or water vapor, hydrophobic PVA-based films can slow food spoilage by stopping water exchange between food and the environment [8,9]. The literature lists a number of techniques for providing packaging materials a hydrophobic property, including combining two or more biopolymers with different properties [10], adding fillers [11,12], applying heat treatment [13], adding plasticizers or cross-linking agents [14,15], adding hydrophobic agents [7,16], etc. In order to prevent water from getting into the food, hydrophobicity primarily involves adding hydrophobic groups, decreasing hydrophilic groups, or compacting the network [17]. By minimizing the transfer of water molecules from the external environment to the food and establishing an effective barrier against moisture, hydrophobic packaging materials are crucial for reducing food waste, minimizing food deterioration, and extending the shelf life of food [18].

Lipids can provide a sufficient barrier against water vapor due to their hydrophobic properties, which make them a good choice for packaging materials. Waxes, resins, paraffins, and acetoglycerides are the lipid materials most frequently utilized to create films or coatings [19]. Waxes are naturally occurring non-polar materials that are highly effective at lowering packaging’s water vapor permeability. Beeswax, candelilla wax, and carnauba wax are the most widely used waxes in the industry. Beeswax is a highly hydrophobic film-forming substance that is naturally produced by bees [1]. It is utilized in a variety of packaging applications, including films or emulsions, composite films, controllable transparency films, active and smart packaging, etc., due to its high ductility, efficiency, and biodegradability [20]. Long-chain hydrocarbons, alcohols, fatty acids, fatty acid esters, free acids, diesters, and other substances make up its composition. It is hydrophobic because 95% of the chain contains methylene carbons [int-(CH2)] [18].

Cheng et al. [7] used the extrusion blowing method to create edible starch and gelatin films with the addition of three types of natural wax: beeswax, candelilla wax, and carnauba wax. They then examined how these waxes affected the physicochemical characteristics of the resulting films. The films’ water resistance and hydrophobicity were enhanced by all kinds of wax, but the beeswax-modified film had the highest hydrophobicity.

Mathew et al. [8] combined cellulose nanofibers and beeswax using the thermally induced phase separation method to create a hydrophobic food packaging material. Beeswax-modified cellulose nanopaper showed a notable decrease in oxygen and water vapor permeability, creating a hydrophobic substance with enormous potential for food packaging. The modified cellulose nanopaper prevented tomatoes from deteriorating and increased their shelf life by up to 15 days; the outcomes were similar to those of tomatoes packaged in commercial LDPE (Low density polyethylene) material.

There is much literature data discussing the inclusion of beeswax in polymeric materials such as starch [7], polyvinyl alcohol [9], ε-polycaprolactone [21], and cellulose [1] in order to achieve a hydrophobic character of the material, suitable as a food packaging material. Almost all the investigated studies related to our study show that the barrier properties of the films increase with the addition of the hydrophobic agent.

In this regard, the novelty of this manuscript relies on establishing the optimal content of BW that can be incorporated into a PVA matrix to obtain the maximum barrier effect against water vapor.

## 2. Materials and Methods

### 2.1. Materials

Poly (vinyl alcohol) (PVA) Mowiol^®^ 28–99, M_w_ = 145,000 g·mol^−1^ was purchased from Sigma-Aldrich^®^ (Merck, Darmstadt, Germany). Glycerin (Gly), citric acid monohydrate (CA), ethanol 96%, and glacial acetic acid were purchased from Chemical Company, Iasi, Romania. Cocamidopropyl betaine extracted from coconut was purchased from Elemental, Oradea, Romania. Beeswax (BW) and castor oil were purchased from a local store with bee and other natural products in Baia Mare, Romania. Beeswax was obtained from a beekeeper in Maramures county, Romania and was purchased in the form of artificial honeycomb (as wax sheet), with a purity of 98%. The melting point was 65 °C. Polyethylene (PE) film used as a reference was purchased from a local supermarket in Baia Mare, Romania.

### 2.2. Development of Films

The solution casting method was used to develop the films. As reference film, the neat PVA was made, by combining 15 g of PVA with 3 wt.% citric acid and 300 mL of surfactant solution (cocamidopropyl betaine extracted from coconut, 10 g/L) in a Berzelius beaker. The mixture was then magnetically stirred at 500 rpm for two hours at 80 °C on a Heidolph MR Hei-End magnetic stirrer in a water bath until a homogenous solution was achieved. An amount of 1.5 wt.% Gly was added at the end, and the film-forming solution was magnetically stirred for an additional ten minutes. The film-forming solution was put into glass Petri dishes with a diameter of 9 cm and left to dry for 48 h at room temperature and on a flat surface. PVA 0 was the code assigned to this film.

The beeswax used was solid and yellow, and was kept at ~22 °C. Solid beeswax was added from the beginning, together with PVA, citric acid, and surfactant solution, in varying mass proportions related to the total mass of the raw materials, and the prepared films were coded as PVA 1 (3.5 wt.% BW), PVA 2 (6.75 wt.% BW), PVA 3 (9.79 wt.% BW), PVA 4 (12.63 wt.% BW), PVA 5 (15.29 wt.% BW), and PVA 6 (17.79 wt.% BW).

All PVA films modified with BW as well the control PVA film were heat-treated at 125 °C for 30 min. Three replicas for each film were prepared.

### 2.3. Characterization of Films

#### 2.3.1. Morphology by Scanning Electron Microscopy (SEM) and Elemental Analysis by Energy-Dispersive X-Ray Spectroscopy (EDXS)

The morphology of the films was determined by scanning electron microscopy, using an FEI Helios NanoLab 650 DualBeam high-resolution microscope (FEI Company (acquired by Thermo Fisher Scientific, Eindhoven, The Netherlands)) that combines a scanning electron microscope (SEM) with a focused ion beam (FIB) for tasks such as nanoscale characterization, prototyping, and sample preparation. The specific configuration includes an Oxford Aztec EDXS system and an X-max silicon drift detector (SDD) with an active surface area of 50 mm^2^ for energy-dispersive X-ray spectroscopy (EDXS), allowing for detailed chemical analysis of materials. The samples were fixed on the SEM sample holder with double-sided tape and coated with carbon (~10 nm) using flash evaporation. The working potential was set to 2 kV for imaging and 15 kV for EDXS analysis.

#### 2.3.2. X-Ray Diffraction (XRD)

A Shimadzu X-ray diffractometer (XRD 6000, Kyoto, Japan) operated with CuKα radiation (λ = 1.54 Å) and a Ni filter was used to investigate the crystallization degree of the films. The diffraction patterns were recorded in a 2θ range from 10° to 80° with a scan speed of 2°/min. The crystallinity rate of the sample was calculated with the following equation:

(1) $$\mathrm{Degree}\;\mathrm{of}\;\mathrm{crystallinity}\;(\%)=\;\frac{I_{\mathrm{crystalline}}}{I_{\mathrm{total}}}100$$

where I_crystalline_ and I_total_ represent the area of the crystalline phases and the total area of the profile, respectively, and were calculated by FullProof Suite software(64-bit Windows). The primary crystallite size of the materials was calculated using the Scherrer equation:

(2) $$D\;=\;\frac{K\lambda }{\beta \mathrm{cosϴ}}$$

where K is a constant with value ~0.89, λ the wavelength, θ the diffraction angle, and β the full width at half maximum (FWHM).

#### 2.3.3. Fourier-Transform Infrared Spectroscopy (FTIR)

FTIR spectra were collected using a PerkinElmer Spectrum BX spectrometer, at a wavenumber range between 4000 and 600 cm^−1^. Ten measurements were averaged to create each FTIR spectrum, at a resolution of 4 cm^−1^. The normalized value of the FTIR spectra was calculated as follows: (experimental value − minimum value)/(maximum value − minimum value). The deconvoluted FTIR spectra were used to quantitatively analyze free hydroxyl groups and intra- and intermolecular hydrogen bonds by allocating the absorption bands in the 3000–3600 cm^−1^ range [22]. The quantity of free hydroxyl groups and intermolecular and intramolecular hydrogen bonds was determined using the ratio of integrated areas, by using the Gaussian mathematical line shape. The Fityk program was used for deconvolution.

#### 2.3.4. X-Ray Photoelectron Spectroscopy (XPS) Analysis

The XPS spectra were obtained using a SPECS PHOIBOS 150 MCD system (Berlin, Germany) equipped with a monochromatic AlKα source (200 W, hν = 1486.6 eV), a hemispherical analyzer, and a multichannel detector. The experiments were conducted by operating an X-ray source with a power of 200 W. The analysis chamber pressure was between 10^−9^ and 10^−10^ mbar. The charge neutralization procedure was used for all samples using a Flood gun component. The binding energy scale was referenced to the carbon component C1s at 284.6 eV. Survey spectra acquired at a pass energy of 60 eV were used to determine the elemental compositions (at%). High-resolution spectra were collected at an analyzer pass energy of 20 eV. The Shirley method was applied for background subtraction in the fitting procedure.

#### 2.3.5. Surface Analysis by Atomic Force Microscopy (AFM)

The AFM images were recorded in non-contact (tapping) mode using a system from Molecular Devices and Tools for Nano Technology (NT-MDT) mounted on an Olympus IX71 optical microscope and acquired as a whole from Spectrum Instruments Ltd. (Limerick, Ireland). The AFM measurements were conducted by employing high-resolution noncontact gold silicon probes from NT-MDT, exhibiting a tip radius of a curvature smaller than 10 nm and a tip height of about 15 ± 1 µm. All probes were also Au-coated on the detector side cantilever. The latter had a length of 125 ± 5 µm and a resonance frequency in the range of 187–230 kHz. Moreover, the nominal force constant of the cantilever ranged between 1.45 and 15.1 N/m. The AFM images (256 × 256 lines) were acquired using a typical scanning speed of about 1–2 µm/s and a setpoint carefully adjusted between 9 and 12 V to maintain a very soft tapping regime.

#### 2.3.6. Differential Thermal Analyses (DTA) and Thermogravimetry (TGA)

Using a thermal analyzer DTG-60H (Shimadzu, Kyoto, Japan), which concurrently records the differential thermal analysis (DTA) curve and thermogravimetric curve (TGA), the thermostability and thermal degradation behavior of the prepared films were evaluated in the temperature range of 30 to 800 °C. The sample was placed in an alumina crucible and covered with a perforated alumina lid, the reference material being α-alumina. The samples were heated in the 30–800 °C temperature range, with a heating rate of 10 °C/min, in a nitrogen atmosphere with a flow rate of 70 mL/min. TA60 software (version 2.20) was used to analyze data.

#### 2.3.7. Water Vapor Transmission Rate (WVTR) and Water Vapor Permeability (WVP)

The film piece was fixed to a plastic 1 cm × 1 cm vial containing anhydrous silica gel (~3 g). The assembly was weighed and then placed in a water vapor container. The assembly was weighed daily, and the WVTR (g/m^2^/24 h) was calculated. The relative humidity inside the water vapor container was 90% and the temperature was 22 °C. The surface of the material exposed to the water vapor was 1 cm^2^.

The WVP was calculated using the formula:

(3) $$\mathrm{WVP}\;(g/s\cdot m\cdot \mathrm{Pa})=\frac{m\cdot x}{t\cdot A\cdot \Delta P}$$

where m—mass increase due to the absorption of water vapor by silica gel until equilibrium is reached (g); x—thickness of the material (m); A—contact surface area (m^2^); t—analysis time required to reach equilibrium (s); ΔP—difference between the partial pressure of the atmosphere with silica gel and distilled water (2652 Pa at 40 °C).

#### 2.3.8. Permeability to Grease

An amount of 2 g of castor oil was inserted into the plastic bottle, and the vial was covered with the film to be analyzed. Subsequently, the vial was turned over onto a piece of filter paper (weighed in advance) and was kept both refrigerated (4 °C) and at room temperature (20 °C) for 24 h. After that, the filter paper was re-weighed, and the grease permeability was determined as the percentage of oil that penetrated the film within 24 h.

(4) $$\mathrm{Permeability}\;\mathrm{to}\;\mathrm{grease}\;\left(\%\right)=\frac{m_{f}-m_{i}}{m_{i}}\;\cdot 100$$

where m_f_—mass of filter paper after 24 h of storage (g); m_i_—the mass of the filter paper at the beginning of the experiment (g).

#### 2.3.9. UV-Blocking Performance

The ultraviolet-visible (UV-Vis) barrier properties of the developed films were evaluated using a PerkinElmer Lambda 35 spectrophotometer within a wavelength range of 200 to 800 nm. The UV-blocking performance of the films was subsequently calculated using the following equation:

(5) $$\mathrm{Opacity}\;\left(u.a.\mathrm{at}\;600\;\mathrm{nm}/\mathrm{mm}\right)=\;\frac{\mathrm{Abs}.\mathrm{at}\;600\;\mathrm{nm}}{x}\;$$

where Abs. at 600 nm—absorbance at 600 nm; x—film thickness (mm).

#### 2.3.10. Solubility and Migration in Food Simulants

The method consists of assessing the solubility of films in simulant A (10 wt.% ethanol solution that mimics aqueous food), simulant B (3 wt.% acetic acid solution that mimics acidic food), and simulant D1 (50 wt.% ethanol solution that mimics fatty food) by gravimetric analysis. Three pieces (1 cm^2^) were cut out of each film and weighed, and were then immersed in 10 mL of simulant. The samples were left at 22 °C and 4 °C for 24 h, and were afterwards removed, dried, and weighed. The solubility was calculated according to the formula:

(6) $$S\;\left(\%\right)=\;\frac{m_{i}-m_{f}}{m_{i}}\;\cdot 100$$

where S—solubility (%); m_i_—the initial mass of film pieces (g); m_f_—the final mass of film pieces (g).

Migration was determined in the same conditions as those used for solubility determination and was expressed in mg/dm^2^ in 24 h, taking into consideration that the material was cut into pieces of 1 cm by 1 cm.

#### 2.3.11. Water Absorption (Equilibrium Water Content—EWC)

The analysis consists in monitoring the film mass before and after immersion in a phosphate buffer solution pH = 7.4, until equilibrium (~3 h) is reached at 37 °C. A piece of film with a known mass was inserted in 10 mL phosphate buffer pH = 7.4 and stored at 30 °C for 3 h. Subsequently, the piece of film was removed, and the excess liquid was absorbed with filter paper and weighed. The EWC was calculated with the formula:

(7) $$\mathrm{EWC}\;\left(\%\right)=\;\frac{m_{f}-m_{i}}{m_{i}}\;\cdot 100\;$$

where m_i_—the initial mass of film pieces (g); m_f_—the final mass of the film piece (g).

#### 2.3.12. Tensile Strength

Tensile strength (TS) was determined as the pressure (kN/m^2^) applied to break a piece of material of 1 cm width and 3 cm length. One side of the material, of 1 cm length, was fixed between two metal plates and attached to a support, and the other side, also of 1 cm length, was fixed between two other metal plates to which a textile container was also attached. A fabric bag was filled with weights until the material broke. The analysis was performed at 22 °C. Tensile strength was calculated as the pressure applied to the material until it broke, and it was calculated using the formula:

(8) $$\mathrm{TS}=\frac{m\;\cdot g}{l\;\cdot h}$$

where m—mass that breaks the material (kg); g—gravitational acceleration (9.81 m/s^2^); l—width of the material (m); h—material thickness (m).

#### 2.3.13. Statistical Analysis

The experiments for the physical–chemical characterization were carried out in three replicates and the results were reported as mean values ± standard deviation. Statistical processing was subsequently performed using IBM SPSS Statistics software (version 24, IBM Corp., Armonk, NY, USA). Group means were compared using one-way Analysis of Variance (ANOVA) followed by Tukey’s test (*p* < 0.05).

## 3. Results and Discussion

### 3.1. Homogeneity of the Films

FTIR analysis was used to evaluate the film’s homogeneity, as follows: FTIR spectra were recorded at ten points from each of the three replicates. The average transmittance and error were computed for each wavenumber. The mean error was calculated by averaging the errors from all wavenumbers. Each PVA-based film’s three-replica FTIR spectra are shown in the Supplementary Materials (Figure S1a–g). This means that the films are homogeneous and that the preparation procedure is repeatable, as indicated by the maximum error obtained in each case, which ranged between 0.27% and 4.13% (Table 1).

### 3.2. Morphology of the Films by Scanning Electron Microscopy (SEM) and Elemental Analysis by Energy-Dispersive X-Ray Spectroscopy (EDXS)

The neat PVA film appeared uniform, compact, flexible, transparent, and colorless (Figure S2a, Supplementary Materials). The PVA-BW films are heat-sealable and the connection is stable. The films modified with BW were homogeneous from a macroscopic point of view, compact, less flexible, and became more opaque as the BW content increased, having a color ranging from pale to a more pronounced yellow (Figure S2b–g—Supplementary Materials). Hromič et al. [24] also reported similar findings. They made chitosan-based films and added BW and cumin essential oil, demonstrating how the lipids altered the composite films’ thickness and color.

The BW dispersion into the PVA matrix was assessed using the morphological analysis of the films (Figure S3a–n, Supplementary Materials). Samples PVA 0, PVA 1, and PVA 2 with BW between 0 wt.% and 6.75 wt.% are shown in Figure S3a–f (Supplementary Materials) and had a uniform dispersion of wax throughout and a smooth, continuous surface with pleated chains that were free of cracks or gaps. As the BW intake increased, so did the surface roughness.

With a higher BW percentage (ranging from 9.79 weight percent to 17.79 weight percent), SEM micrographs of PVA 3, PVA 4, PVA 5, and PVA 6 films (Figure S3g–n, Supplementary Materials) showed a rougher, granular, but crackless surface, typical of wax-induced topography, and small darker portions, indicating phase separation [25]. BW can be identified in the matrix by the lighter areas—yellowish-white protrusions with a scaly appearance. An increase in the extent of agglomeration was observed upon increasing the BW content within the matrix [26,27]. Regions characterized by a lower wax content, corresponding to the darker domains, may compromise the film’s water vapor barrier efficiency, thereby facilitating the diffusion of water molecules through the matrix. Similar results were reported by Qamar et al. [25], who obtained functional films from carboxymethylcellulose. They used cellulose extracted from cocoa husks, which was plasticized with glycerol or sorbitol and treated with natural waxes (BW and carnauba wax). They reported rougher and more uneven surfaces for films containing BW, specific to the wax-induced topography. Huang et al. [28] also reported the preparation of hydrophobic chitosan and PVA films by spraying with a solution of BW and SiO_2_, and found that the film structure showed unevenness and gaps at the nanoscale. Diyana et al. [29] observed that an increase in BW content in glycerol-plasticized starch films induced gaps in the microstructure, caused by different polarity between hydrophilic starch and hydrophobic wax, as well as by starch degradation and wax interaction with plasticizer, leading to phase separation.

EDXS quantitative analysis was conducted to elucidate the distribution of BW in the bulk PVA. The presence of carbon at a percentage higher than 60 wt.% and oxygen at lower than 40 wt.% was noticed. The carbon content in the neat PVA (PVA 0) is 60 (atom %) and rises as the BW is added up to 77 (atom %). The carbon content increases with the increase in the mass of BW added, as expected, due to the large number of hydrocarbon chains in the wax constitution [28]. Elemental analysis revealed a direct correlation between BW content and carbon concentration. As detailed in Table 1, the subsequent decline in the O/C ratio confirms the surface accumulation of the wax layer. This migration lowers the film’s surface polarity and stems from the competitive interactions between the hydrophilic groups of PVA and hydrophobic groups of BW. Additionally, there is a difference in the elemental content between the bulk and the surface, which increases as the BW is added, demonstrating again the agglomeration of BW on the surface, results confirmed by the SEM images. Ponnusamy et al. [30] reported that a high proportion of carbon in slow-release, nano-biocarbon-coated fertilizers derived from sugarcane bagasse and polymers such as PVA and polyvinylpyrrolidone (PVP) is due to the formation of stable structures, which provide mechanical strength and long-term stability.

### 3.3. Structure by X-Ray Diffraction (XRD) and Fourier-Transform Infrared Spectroscopy (FTIR)

**Figure 1:**
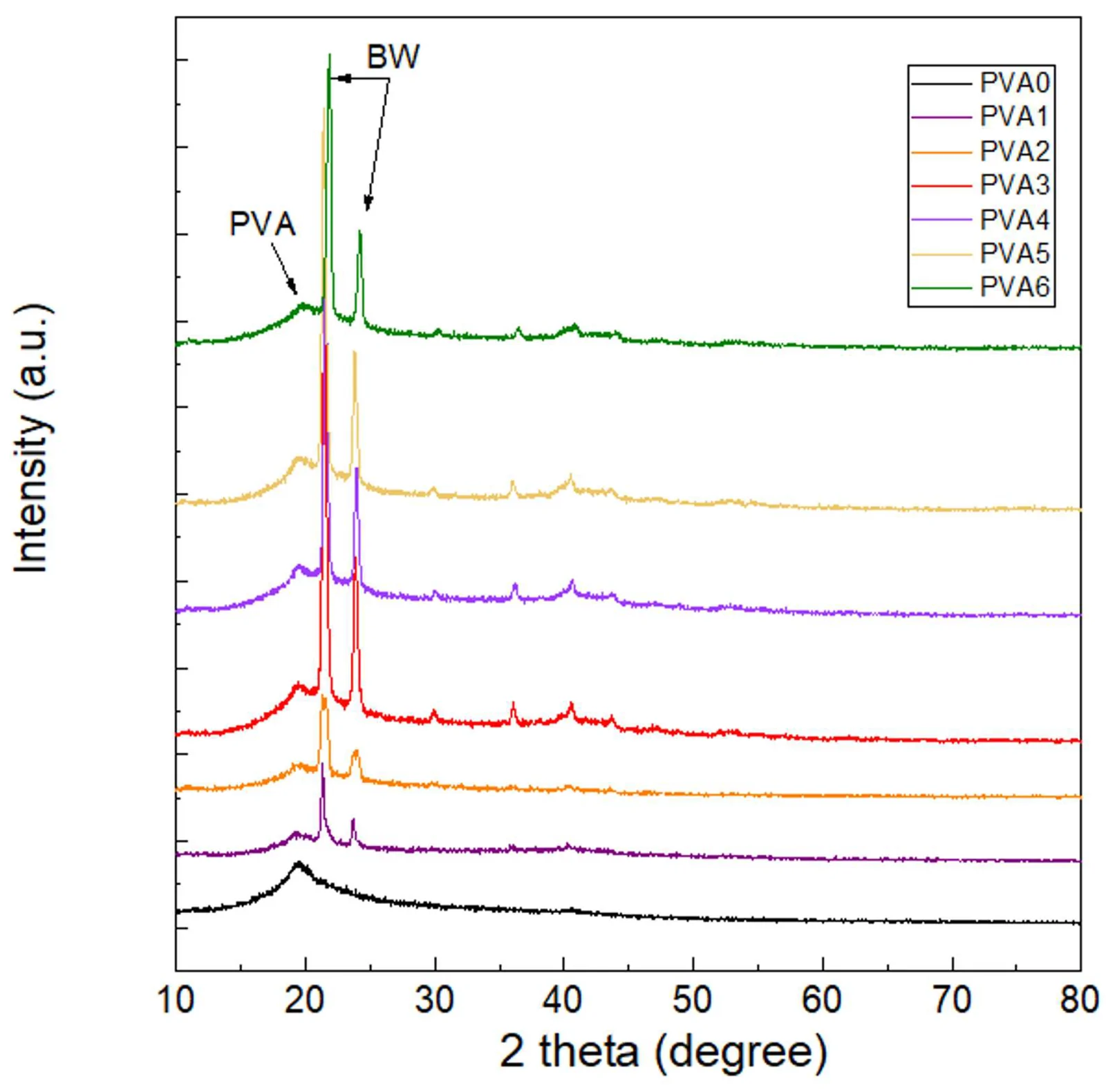
XRD patterns of PVA-based films (PVA 0 (0 wt.% BW), PVA 1 (3.5 wt.% BW), PVA 2 (6.75 wt.% BW), PVA 3 (6.75 wt.% BW), PVA 4 (6.75 wt.% BW), PVA 5 (6.75 wt.% BW) and PVA 6 (6.75 wt.% BW)).

As illustrated in Figure 1, structural modifications within the crystalline or intercalated phases are clearly evidenced by shifts and intensity alterations in the XRD diffraction patterns.

The neat PVA film exhibits a predominantly amorphous matrix characterized by a broad, low-intensity scattering region, alongside a distinct crystalline reflection at 2θ = 19.7° corresponding to the (101) lattice plane [1,31]. This structural profile corresponds to a minimal degree of crystallinity, of only 0.52%. The modified films showed well-defined diffraction peaks, specific to BW crystals at 2θ = 21.4° and 2θ = 23.8°, corresponding to hydrocarbon and monoester fractions, which tend to crystallize in an orthorhombic structure [32,33]. The degree of crystallinity within the composite PVA-BW matrix (Table 2) demonstrated a positive correlation with BW content up to a threshold of 9.79 wt.% (PVA 3), suggesting that the BW presence did not alter the PVA organization. However, the crystallinity of PVA 4 and PVA 5 (with 12.63 and 15.29 wt.% BW, respectively) was lower than that of PVA 2 and PVA 3 because of the PVA arrangement perturbation assigned by BW, demonstrating the interactions between the compounds from BW and PVA chain. Zhang et al. [33] indicated that these reflections correspond to the short distances between the fatty acid chains (4,2 Å and 3,8 Å, respectively), associated with methylene groups in the hydrocarbon chain of BW. The crystallinity of PVA 6 (17.79 wt.% BW) is similar to that of PVA 3, denoting a separation of PVA and BW phases. PVA 1 film contains mean crystallites with the largest dimensions and the lowest degree of crystallinity from all samples, indicating a limited nucleation process, in which few crystalline domains were formed, but they were able to grow extensively in a predominantly amorphous matrix. These findings align with the structural insights provided by FTIR spectroscopy and support the data regarding the water vapor barrier properties (Section 3.7). Similar results were obtained by Mo et al. [34], who prepared PVA films, plasticized with acetamide and lignin as a secondary green plasticizer, and showed that the increase in the amount of lignin reduced the nucleation and increased the particle size. Similarly, Mathew et al. [8] demonstrated that the incorporation of BW into cellulose nanopaper enhanced the overall degree of crystallinity of the resulting composite.

**Figure 2:**
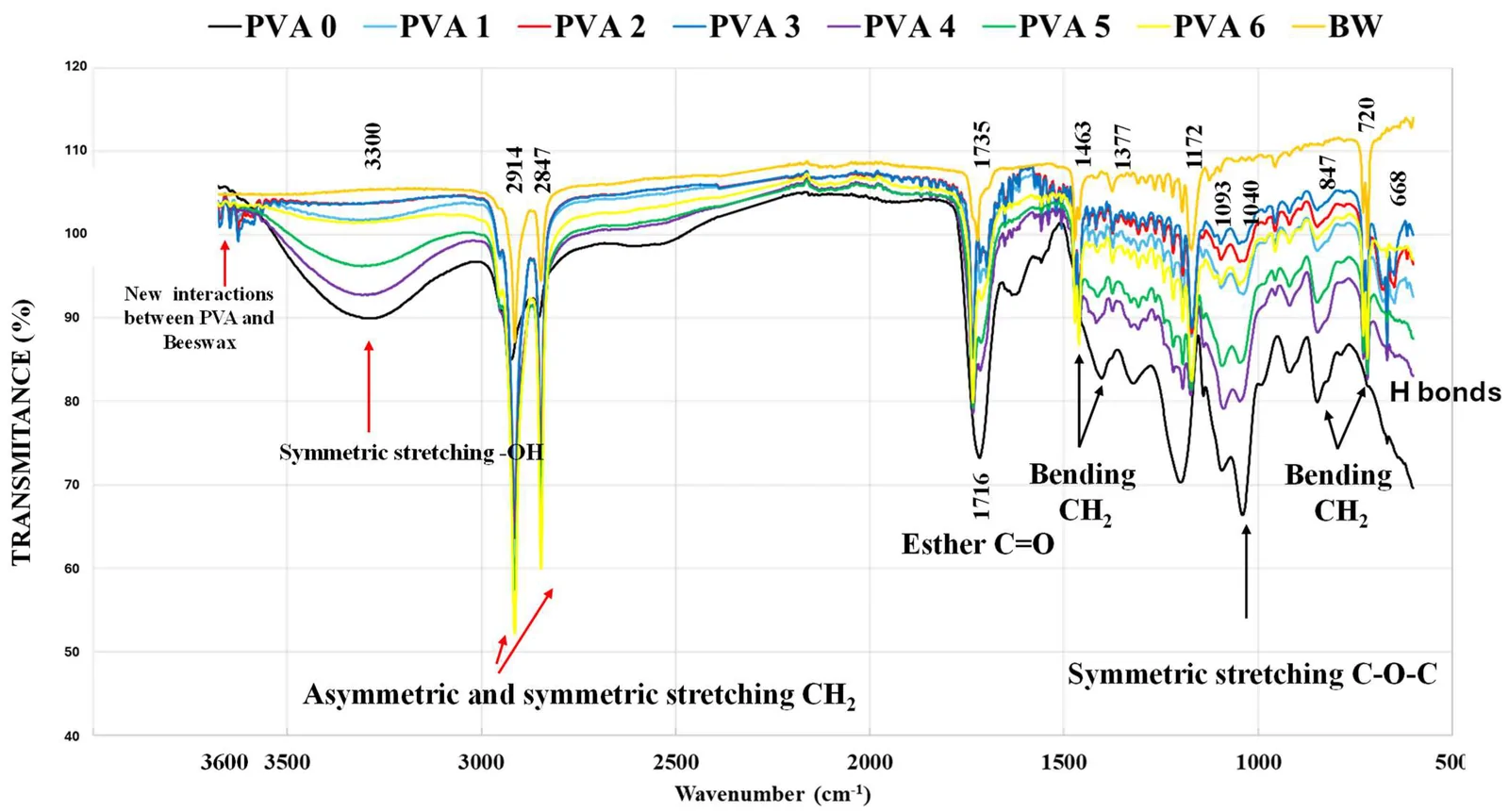
FTIR spectra of the PVA films (PVA 0 (0 wt.% BW), PVA 1 (3.5 wt.% BW), PVA 2 (6.75 wt.% BW), PVA 3 (6.75 wt.% BW), PVA 4 (6.75 wt.% BW), PVA 5 (6.75 wt.% BW) and PVA 6 (6.75 wt.% BW)).

FTIR analysis was performed to investigate the chemical modifications induced within the PVA matrix upon BW incorporation. The FTIR spectra are illustrated in Figure 2. The absorption bands observed in the 3600–3567 cm^−1^ region are uniquely discernible in samples PVA 1–PVA 5, while remaining entirely absent in the spectra of PVA 0, neat BW, and PVA 6 [28]. The absence of these signals from PVA 6 shows that 17.79 wt.% BW in the matrix is excessive and induces phase separation, behavior confirmed also by SEM. The signal in the range 3200–3650 cm^−1^ is a convoluted signal resulting from free OH groups (3580–3650 cm^−1^), intramolecular hydrogen bonds (3400–3550 cm^−1^), and intermolecular hydrogen bonds (3200–3400 cm^−1^) [33]. The intensity of this signal decreases in the following order:

PVA 0 > PVA 4 > PVA 5 > PVA 1 > PVA 6 > PVA 3 > PVA 2 > BW

These results demonstrate the near-absence or negligible density of hydroxyl (–OH) groups in neat BW, alongside their substantial depletion within the PVA 3 matrix. This reduction is attributed to etherification reactions with the hydroxyl groups of glycerin (Gly) and/or esterification with the carboxyl groups derived from either citric acid or the fatty acid constituents of BW. Consequently, the most extensive crosslinking network was achieved in samples PVA 3 and PVA 1 [28]. The evolution of the iterative spectral deconvolution process for free OH and intra- and intermolecular H bonds at 3580–3650 cm^−1^, 3400–3550 cm^−1^, and 3200–3400 cm^−1^, respectively, is illustrated in Supplementary Materials—Figure S4, and the preponderance of free -OH and intra- and intermolecular H bonds is included in Table 3. The share of polymer–polymer intermolecular hydrogen bonds decreases as the BW content increases from PVA 0 to PVA 5 (0 wt.% to 15.29 wt.%), because the long chains of hydrocarbons and palmitic esters in BW generate a steric impediment and physically intercalate between PVA chains, spacing them apart and disrupting the initial PVA arrangement. This phenomenon also partially reduces the overall degree of crystallinity of the polymer matrix, as confirmed by the XRD results. The share of intramolecular hydrogen bonds increases as the BW content increases from PVA 0 to PVA 5 (0 wt.% to 15.29 wt.%), because the neighboring polymer chains are far apart by the BW phases, -OH groups on the same chain seeking the energy stability locally. The PVA chain segment, no longer having a correspondent on an adjacent chain (due to the hydrophobic barriers introduced by the BW), tends to form hydrogen bonds with the -OH groups on the same molecule. The free OH groups increase with the BW content from PVA 0 to PVA 5 (0 wt.% to 15.29 wt.%), due to the phase separation between BW and PVA. Thus, many -OH groups of PVA remain isolated at the interface between the hydrophilic matrix and the BW [35]. These variations do not extend to sample PVA 6, where the density of intermolecular hydrogen bonding exceeds that of PVA 5, while the percentages of intramolecular hydrogen bonds and free –OH groups decrease. This divergence indicates localized phase separation between the PVA matrix and the BW phase, which is validated by XRD and SEM characterizations.

The band at 1716 cm^−1^ in the spectrum of PVA 0 is assigned to the carbonyl (C=O) stretching vibration of ester groups, providing further evidence that esterification occurred between the hydroxyl functionalities of PVA and the carboxyl groups of citric acid. This signal is misplaced at 1734 cm^−1^ in BW and at 1735 cm^−1^ in the waxed films, suggesting that esterification also involved the fatty acid fractions of the BW. The signal at 1463 cm^−1^ appears exclusively in the waxed films and is assigned to irregular deformation of C-H bonds and -OH groups from PVA as a result of the beeswax [28]. All FTIR spectra contain a band around 1460 cm^−1^ assigned to the bending of C-H [28]. The signals between 800 and 1400 cm^−1^ are assigned to the vibration of C-C bonds (at 718 cm^−1^ from BW, between 500 and 782 cm^−1^ from PVA), the bending of methylene groups, and hydrogen bonds [28].

The band at 1090 cm^−1^ is assigned to the stretching of C-O-C bonds and appears in all FTIR spectra, indicating the etherification between -OH groups from PVA, Gly, and superior alcohols from BW. PVA 4 and PVA 5 have the strongest signal, at 1090 cm^−1^. The signal at 668 cm^−1^ is exclusively detected in the waxed films and is assigned to the conformational changes or secondary interactions, affected by hydrogen bonding [28,36]. This band is weak in PVA 6, suggesting again the phase separation, thus confirming the SEM results.

### 3.4. X-Ray Photoelectron Spectroscopy (XPS) Analysis

XPS analysis was performed for a deeper understanding of the interaction of beeswax with the PVA matrix and the presence of BW on the surface. Deconvolution of C1s and O1s XPS spectra of PVA 0- PVA 6 films is illustrated in Supplementary Materials—Figure S5.

The deconvoluted data of C1s, presented in Table 4, indicated four types of carbon species around 284.6 eV and 286 eV which could be attributed to the C-C/C-H, C-OH/C-O-C, N-C, N^+^-C, and C-OH/C-O-C groups, respectively; in the region from 286.3–287 eV, the C-N^+^ bond is located. The peak at 287.4 eV corresponds to either C=O or C-N bonds, whereas that at 288.9 eV originates from O-C=O/COOH [27]. The magnitude of the peaks C1 and C2 indicates the hydrophobicity of the surface of the PVA films modified with BW. A decrease in the C2 component attributed to the C-O bonds, which also includes the primary hydrophilic group -OH in PVA, and an increase in the C1 component attributed to the C-C bonds, which include aliphatic chains and wax, leads to a higher value of the C1/C2 ratio, which indicates a more hydrophobic surface of these materials. The C1/C2 values of the PVA-BW films were significantly (*p* < 0.05) higher compared to the control (PVA 0) film. Additionally, the deconvoluted data of C1s also show the interconnection between PVA and BW, which occurs in PVA3, PVA 4, PVA 5, and PVA 6 films. Reduced signals around 287 eV are specific for the C-N and C-N^+^ groups [37] from the cocamidopropyl betaine used as surfactant. By integrating the datasets obtained from FTIR and XPS characterization, it can be inferred that while phase separation is pronounced in the PVA 6 formulation (17.79 wt.% BW), with individual PVA and BW domains dominating the morphology, localized microdomains of molecular interconnectivity between the two phases persist, argued by the similar results of free OH groups and intra- and intermolecular hydrogen bonds between PVA 1 and PVA 6. The absence of a discernible IR absorption band for these features indicates that their relative abundance is significantly lower than that observed in the other BW-modified PVA formulations.

The deconvoluted data of O1s, presented in Table 5, indicated two types of oxygen species, namely, carbonyl oxygen assigned to the O-(C=O)-C group (C=O from esters), around 531.8 eV, and alcoholic oxygen attributed to the C-OH/C-O-C/O-(C=O)-C groups (C-O, from PVA), around 533 eV [27]. The binding energy corresponding to the carbonyl oxygen within the ester linkage O-(C=O*)-C shifted from 531.8 eV in the neat PVA 0 control to 531.9 eV and 531.4 eV across the wax-modified composites, aligning with shifts observed in similar systems [34]. An exception to this trend was noted for samples PVA 1 and PVA 6, which exhibited significantly lower signal intensities. Similarly, the photoemission peak for the C-OH/C-O-C/O-(C=O)-C groups shifted from the baseline value of 533.0 eV (PVA 0) to 533.1 eV and 533.6 eV upon wax modification, while demonstrating a general increase in peak intensity across all formulations except PVA 1. The slight shift in the binding energy towards higher values (+0.1 eV) suggests that BW partially covers the surface, creating a more hydrophobic environment, reducing the shielding effect of electrons in the hydroxyl groups of PVA. In contrast, the shift in the binding energy to lower values (−0.4 eV) can be attributed to the formation of hydrogen bonds between the hydroxyl group of PVA and the carbonyl groups (C=O) from BW, suggesting physical interfacial interactions between phases, leading to the compatibility of PVA-wax matrices [34]. This dual behavior may be due to local differences in phase distribution and different wax migration to the surface in the process of film formation [38]. Similarly, Mo et al. [34] demonstrated that introducing lignin into a PVA matrix induced a positive binding energy shift for C-O species in the C 1s region and a negative shift in the O 1s spectra, attributing these electronic variations to strong hydrogen bonding interactions.

The deconvoluted data of N1s, presented in Table 5, indicated two types of nitrogen species from the cocamidopropyl betaine used as surfactant, namely C-N, around 399.5 eV and protonated nitrogen, around 402 eV [37]. The density of these bonds is lower as compared to the others.

The elemental composition at the surface (Table 1) indicates an increase in carbon and a decrease in oxygen with increasing BW content. The C/O ratio increases significantly upon BW incorporation, due to the presence of a large amount of C-H/C-C bonds in PVA-BW, suggesting a migration of the hydrophobic phase to the surface, partially covering the polar groups of PVA. This data positively correlates with the EDXS quantitative elemental analysis.

### 3.5. Surface Analysis by Atomic Force Microscopy (AFM)

**Figure 3:**
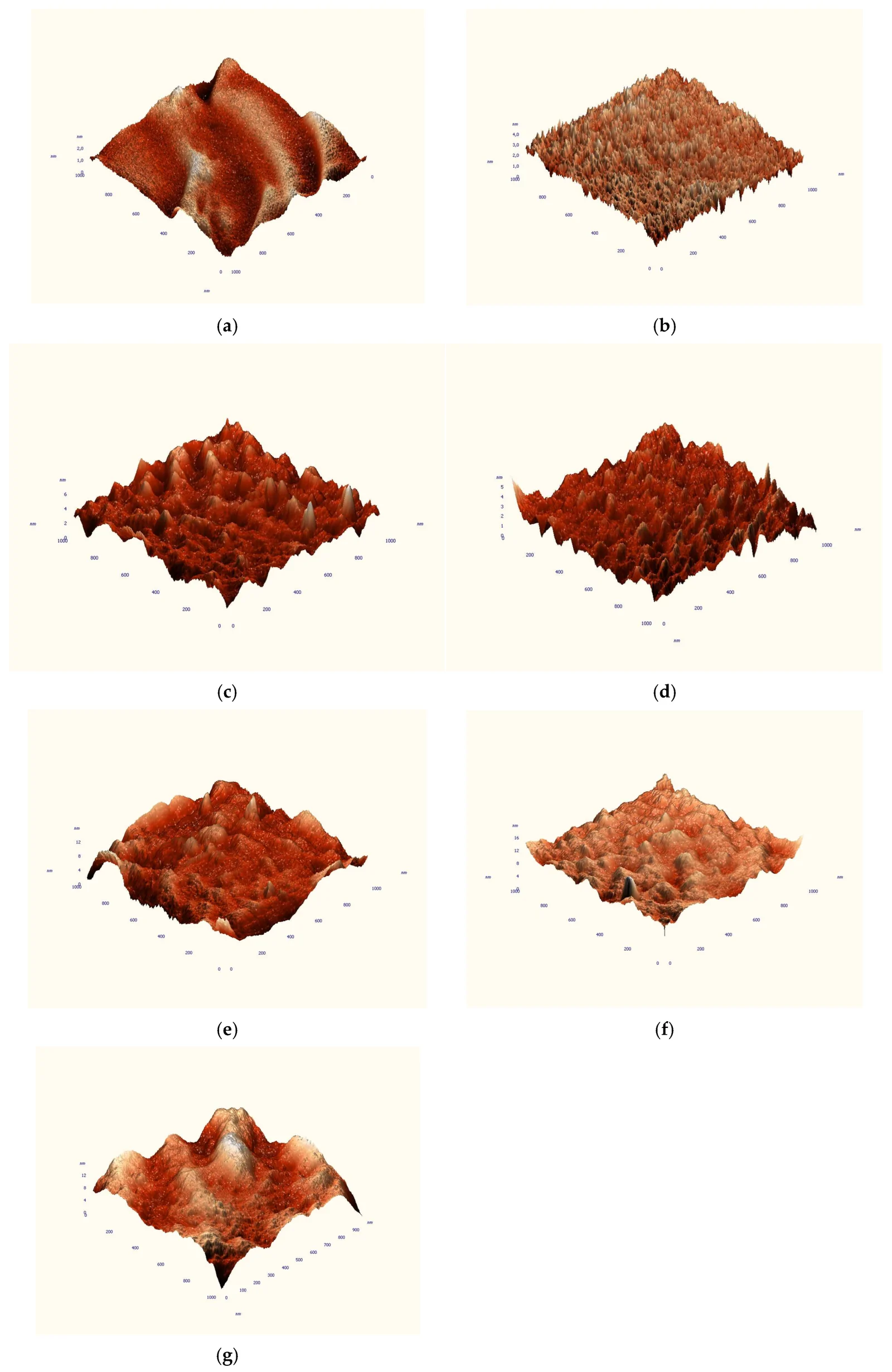
3D images at 1 × 1 μm^2^ magnification of the films: PVA 0 (0 wt.% BW) (**a**), PVA 1 (3.5 wt.% BW) (**b**), PVA 2 (6.75 wt.% BW) (**c**), PVA 3 (6.75 wt.% BW) (**d**), PVA 4 (6.75 wt.% BW) (**e**), PVA 5 (6.75 wt.% BW) (**f**), and PVA 6 (6.75 wt.% BW) (**g**).

The three-dimensional maps resulting from the AFM analysis indicated relatively small differences between the reference PVA 0 film and the films with a low BW content. PVA 0 and PVA 1 showed flatter structures, while PVA 3 and PVA 4 formed rather small structures (Figure 3). The mean and square roughness, as well as the particle size, were determined on topographic images of 1 × 1 μm^2^ and 10 × 10 μm^2^, and are shown in Table 6. The average and square roughness increased proportionally with the amount of BW added, with the exception of the PVA 3 film, which exhibited lower values compared to PVA 2. A similar trend was observed for particle size; however, the surface structures consisted of both larger particles and, frequently, smaller particles. The data extracted from the AFM images confirmed the reproducibility of the samples. Overall, these results are consistent with the SEM observations, confirming the increase in surface roughness with increasing BW content. Similar results were obtained by Ahn et al. [21], who prepared PVA films by electrospinning with BW-modified polycaprolactone nanofibers and found that the surface roughness increased proportionally to BW, but when the amount of BW exceeded 3 g, the surface roughness of the composite film decreased. Increasing surface roughness can lead to improved hydrophobicity of PVA films, according to the Lotus effect, so modified PCL nanofibers can enhance the hydrophobic character of the PVA surface.

### 3.6. Differential Thermal Analyses (DTA) and Thermogravimetry (TGA)

**Figure 4:**
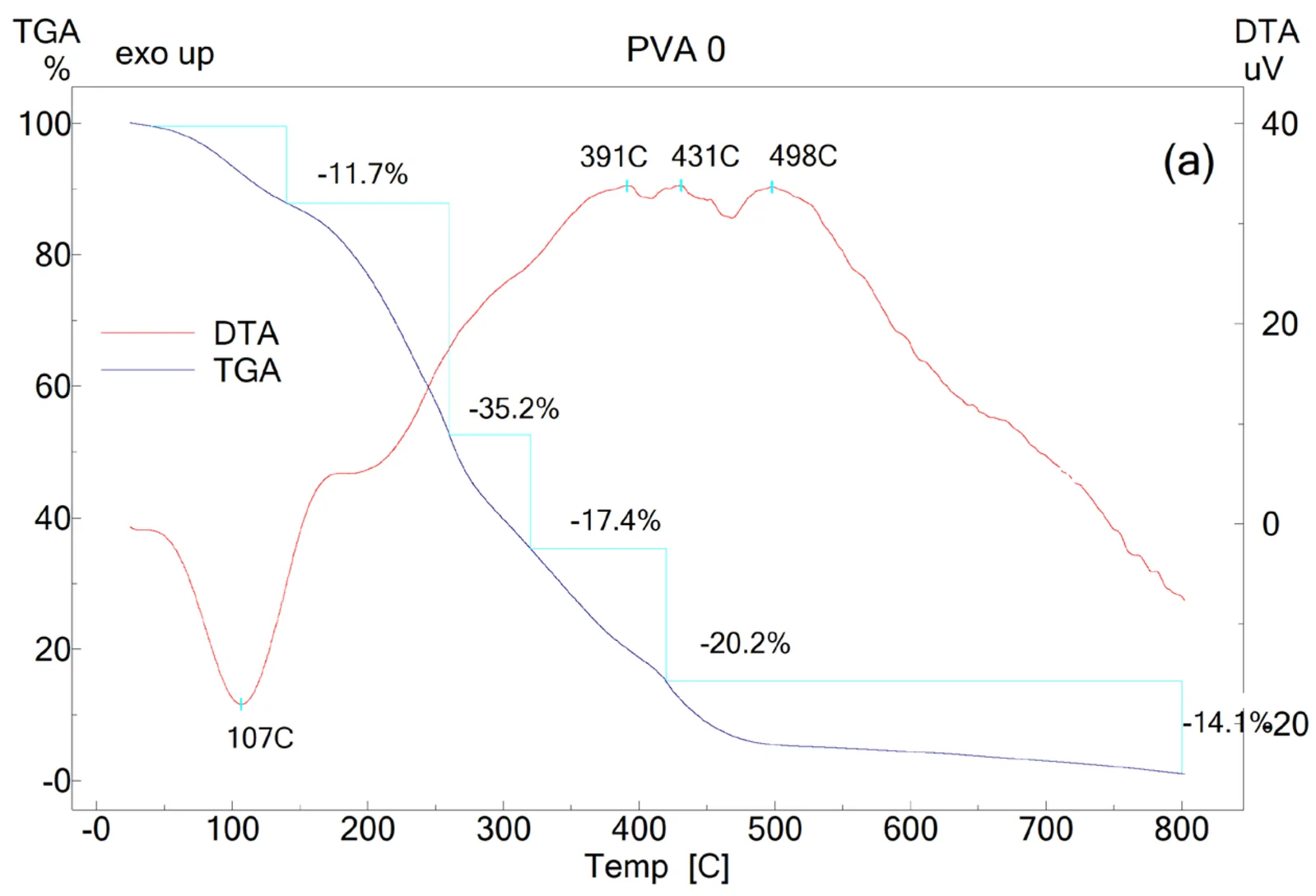

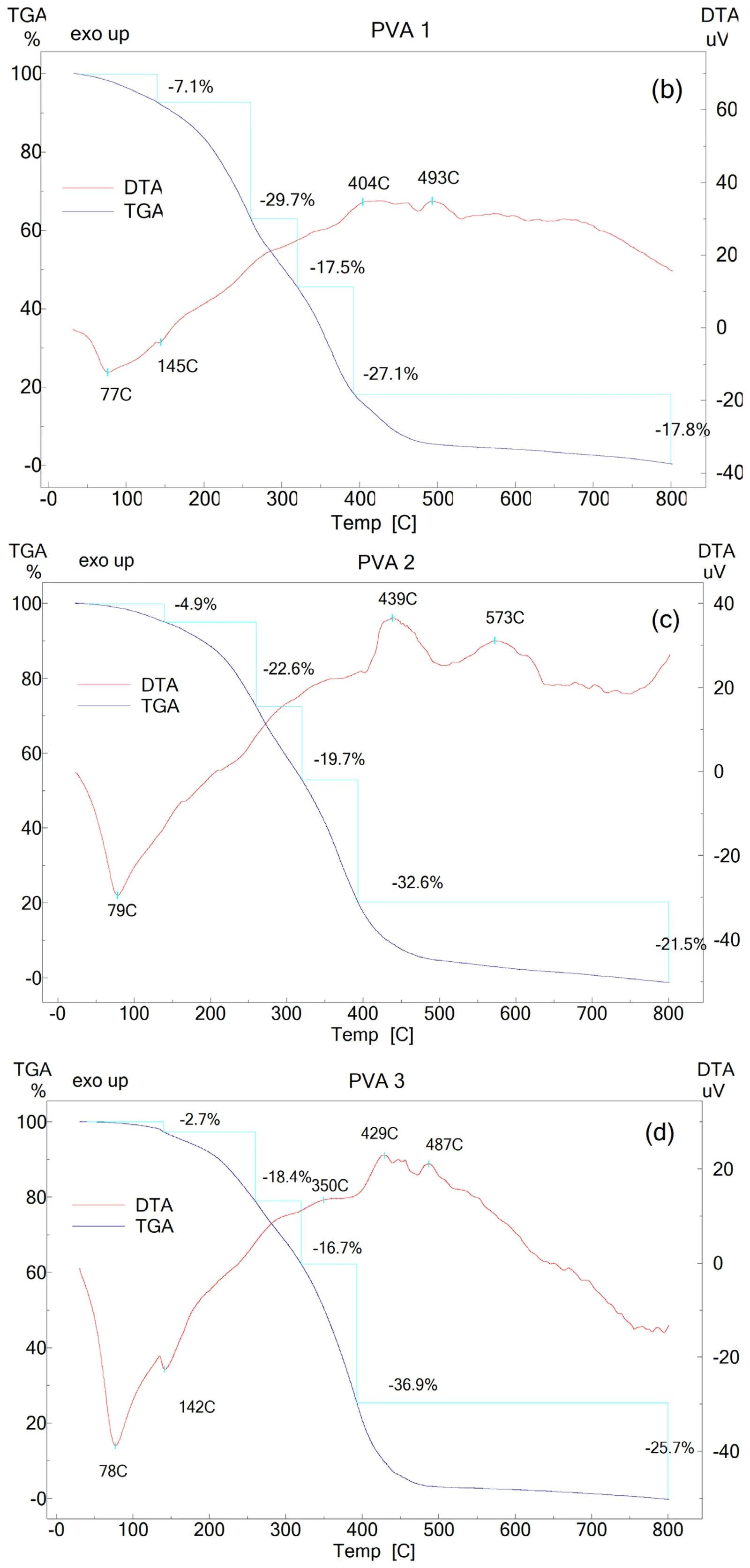

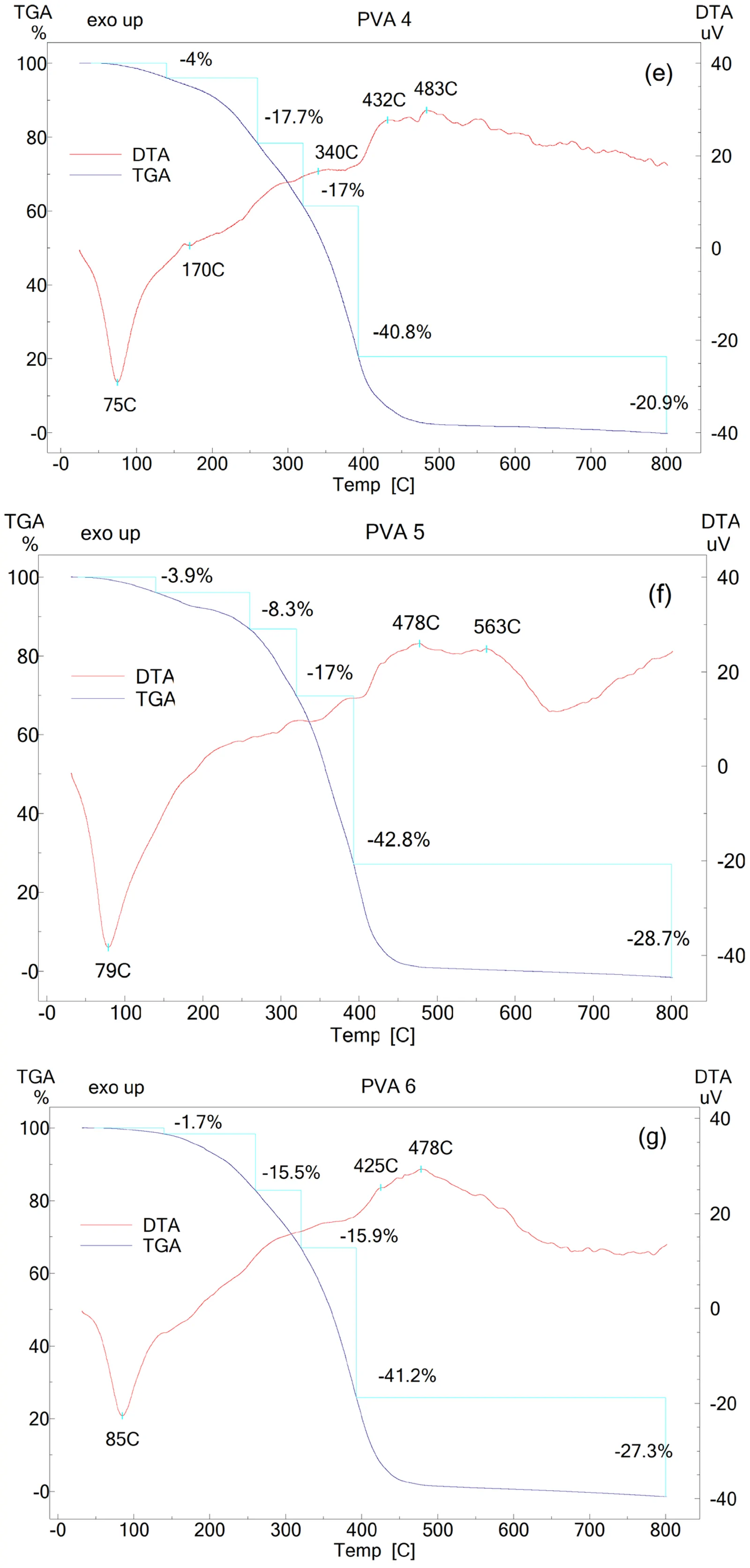
The DTA differential thermal curves and TGA thermograms of the prepared polymer films: PVA 0 (0 wt.% BW) (**a**), PVA 1 (3.5 wt.% BW) (**b**), PVA 2 (6.75 wt.% BW) (**c**), PVA 3 (6.75 wt.% BW) (**d**), PVA 4 (6.75 wt.% BW) (**e**), PVA 5 (6.75 wt.% BW) (**f**), and PVA 6 (6.75 wt.% BW) (**g**).

Simultaneous differential and thermogravimetric analysis (DTA and TGA) were used to determine the characteristic peak temperatures associated with thermal events and to quantify the mass losses of PVA films containing different concentrations of BW. The thermal behavior of PVA and PVA-BW systems is illustrated by the DTA curves and TG thermograms presented in Figure 4. The quantification of the thermal parameters is synthetized in Table 7.

The DTA curves exhibit endothermic peaks between ~75 °C and 85 °C, near the melting point of PVA and BW, respectively [33]. The second thermal event corresponds to BW degradation, with mass losses ranging from ~29.7% to 8.3%, decreasing as BW content increases. Endothermic peaks associated with glycerol vaporization and decomposition are observed in all TGA profiles PVA 0 (107.5 °C), PVA 1 (145 °C), PVA 2 (150 °C), PVA 3 (142 °C), PVA 4 (170 °C), PVA 5 (172 °C), and PVA 6 (175 °C) [1]. The third stage, assigned to polymer chain scission and decomposition, results in mass losses of 15.9–19.7% and exothermic peaks at 350 °C (PVA 3) and 340 °C (PVA 4), values lower than those recorded for PVA 0. In the final degradation stage, all BW-modified PVA films display two well-defined exothermic events, each accompanied by substantial mass losses that reflect the advanced breakdown of the polymer–wax matrix. The onset and intensity of these thermal signals vary across formulations. PVA 1 decomposes through exothermic peaks at approximately 404 °C and 493 °C, with a cumulative mass loss of about 44.9%. PVA 2 shows a more pronounced thermal response, with peaks at ~439 °C and 573 °C and a total mass loss of ~54.1%. For PVA 3, the degradation occurs at ~429 °C and 487 °C, accompanied by a higher mass loss of ~62.6%, while PVA 4 exhibits similar behavior, with peaks at ~432 °C and 483 °C and a mass loss of ~61.7%.

The most extensive degradation is observed in PVA 5, which presents exothermic maxima at ~478 °C and 563 °C and a mass loss of ~71.5%. PVA 6 follows closely, with peaks at ~425 °C and 478 °C and a mass loss of ~68.5%. These results collectively indicate that BW incorporation increased the high-temperature degradation pathway of PVA, leading to more substantial mass losses, but without compromising the overall thermal stability of the films. Based on the thermal analysis, it can be stated that the incorporation of BW does not compromise the thermal stability of PVA films; on the contrary, in certain formulations it contributes to its improvement. The reduced mass losses observed in the dehydration stage confirm the hydrophobic character of BW and its ability to limit moisture retention within the polymer matrix [29,39]. Moreover, the higher degradation temperatures recorded for PVA 2 and PVA 5 compared to neat PVA indicate a BW-induced thermal stabilization, likely associated with the formation of a more compact structure and reduced chain mobility. This behavior is consistent with previous reports showing that the addition of BW to polymeric or cellulosic systems enhances their thermal resistance [1,26]. Overall, the results demonstrate that BW acts as a compatibilizing and stabilizing agent, without inducing adverse effects on the thermal behavior of PVA films.

### 3.7. Water Vapor Transmission Rate (WVTR) and Water Vapor Permeability (WVP)

The WVTR and WVP are parameters reflecting the transfer of water vapor between food and its environment, this being a very important feature affecting the shelf life of a food [40]. The results obtained for the PVA-based films modified with BW were compared to the WVTR values of the neat PVA and the polyethylene (PE) film (Table 8). The best results were recorded for PVA 4 and PVA 5 films registering WVTR values of 103.6 ± 17.7 g/m^2^ × 24 h and 82.5 ± 7.7 g/m^2^ × 24 h, respectively, followed by PVA 6 (186.6 ± 26.8 g/m^2^ × 24 h). The incorporation of 3.5 wt.% BW into the PVA film induced a nearly five-fold reduction in the water vapor transmission rate. An amount between 3.5 wt.% and 9.79 wt.% BW does not significantly modify the WVTR (*p* < 0.05); however, increasing the BW concentration to the range of 12.63–15.29 wt.% yielded an approximately fourfold reduction in the WVTR compared to the formulation containing only 3.5 wt.% BW. The WVTR doubled by increasing the BW mass from 15.29 wt.% to 17.79 wt.%. Increasing the BW content to the range of 12.63–15.29 wt.% yielded a nearly 24-fold enhancement in the moisture barrier capability of PVA, restricting the WVTR to a level just twice that of pure PE. The same variation can be observed also in the case of the WVP as for the WVTR. The WVP for PVA 5 was significantly the lowest from all the tested PVA-based samples.

The results correlate with the morpho-structural analyses (SEM, XRD, and the values of the share of hydrogen and free -OH bonds obtained from the FTIR deconvolution). A BW content of maximum 9.79 wt.% is too low to cause a PVA network disorder (result confirmed by XRD) and to induce a maximum barrier effect against water vapors. A BW content higher than 17.79 wt.% is too high and creates phase separation between PVA and BW. The optimal BW content is between 12.63 wt.% and 15.29 wt.%, providing the maximum barrier against water vapor among all samples developed in the study.

Similar results were obtained by Zhang et al. [40], who prepared agar/maltodextrin (A/M) emulsion films with the addition of three hydrophobic agents (beeswax, refined vegetable oil, and paraffin) and analyzed their effect on the physicochemical properties of the films. The WVP of the A/M decreases if the quantity of beeswax is increased by 10%, but the WVP of the film containing 20% wax is higher than for the film containing 10% wax. The authors suggested that exceeding an optimal threshold of the hydrophobic agent could increase the WVP of the reference film due to network congestion.

### 3.8. Permeability to Grease

Evaluating grease permeability is essential for food packaging design, as it dictates the material’s efficiency in preventing oil and fat permeation across the film structure. Low fat permeability can prevent spoilage of packaged products and loss of food quality and safety. The fat permeability of the obtained films was tested against castor oil, and the results obtained demonstrated the impermeability of the films, both at 4 °C and 22 °C. PVA-based films modified with BW could represent a potential food packaging for foods with a high fat content.

### 3.9. UV-Blocking Performance

**Figure 5:**
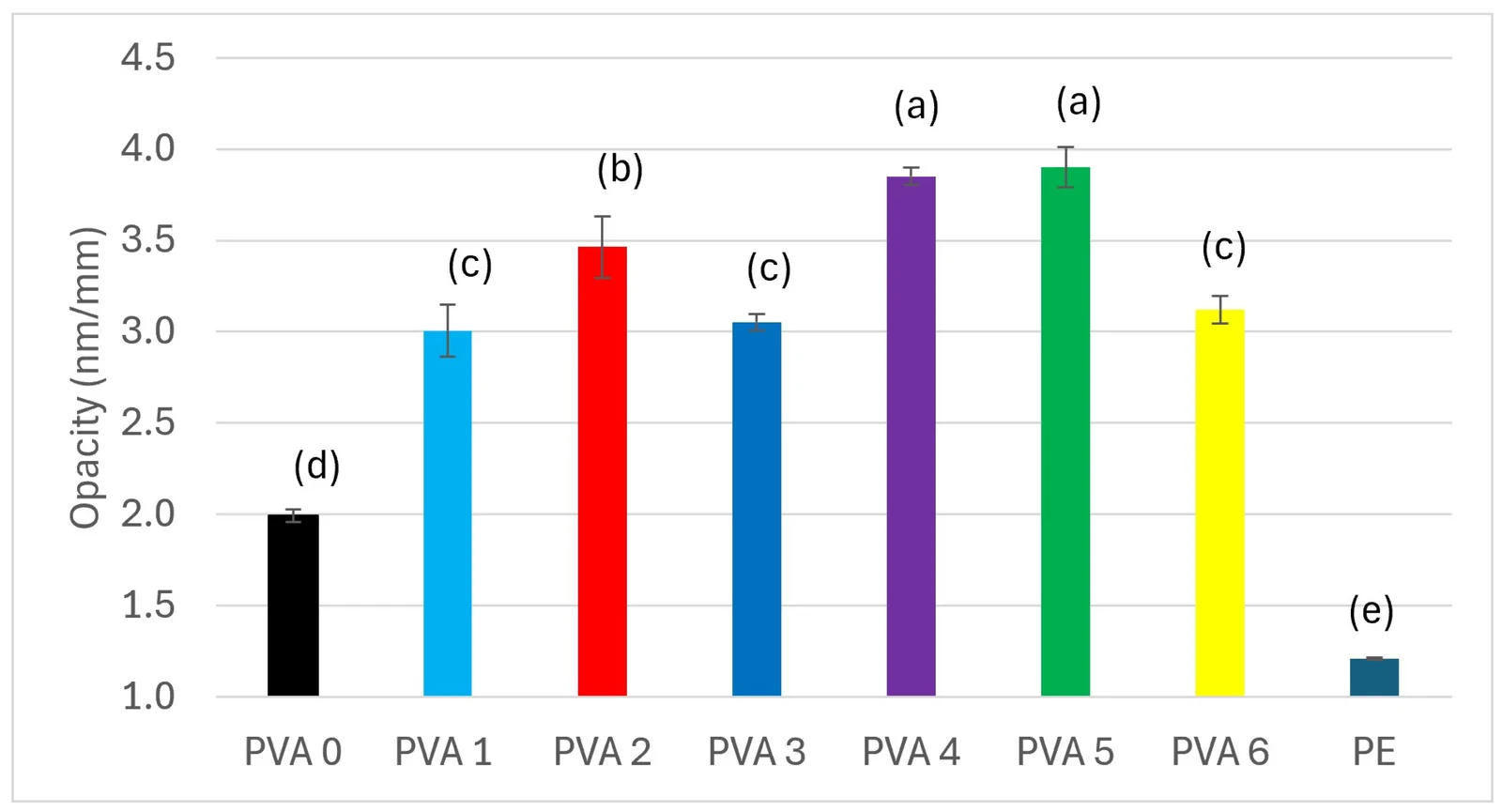
The UV-blocking performance of the films PVA 0 (0 wt.% BW), PVA 1 (3.5 wt.% BW), PVA 2 (6.75 wt.% BW), PVA 3 (6.75 wt.% BW), PVA 4 (6.75 wt.% BW), PVA 5 (6.75 wt.% BW), and PVA 6 (6.75 wt.% BW) (PE—polyethylene) (the same letter means no difference in statistical significance between the values; different letters means that the difference between values is statistically significant (*p* > 0.05, according to one-way ANOVA and the Tukey model)).

Proper UV-Vis light protection of a packaging material can prevent lipid oxidation and food degradation, thus avoiding food waste and extending the shelf life of food. The UV-blocking performance values of the films obtained are presented in Figure 5. All PVA films modified with BW improved their barrier properties against UV radiation compared to the control (PVA 0). Increasing the hydrophobic agent content progressively elevates film UV-blocking performance, which precisely doubles at a BW loading range of 12.63–15.259 wt.%. Above this value (PVA 6), the UV-blocking performance decreases due to phase separation, as confirmed by SEM, XRD, FTIR, and XPS. Ariati et al. [20] showed that visible domain transmittance values decrease at room temperature as BW is added in different mass proportions to polydimethylsiloxane-based films. Also, Oliveira et al. [41] indicated a more yellowish color of the cassava starch, corn starch, and gelatin-based composite with the introduction of BW into the composite, with the aim of improving the barrier performance of the composite, thereby increasing the UV-blocking performance of the film. The film thus obtained proved its effectiveness in preserving guava fruits and extending their shelf life.

### 3.10. Solubility and Migration in Food Simulants

**Figure 6:**
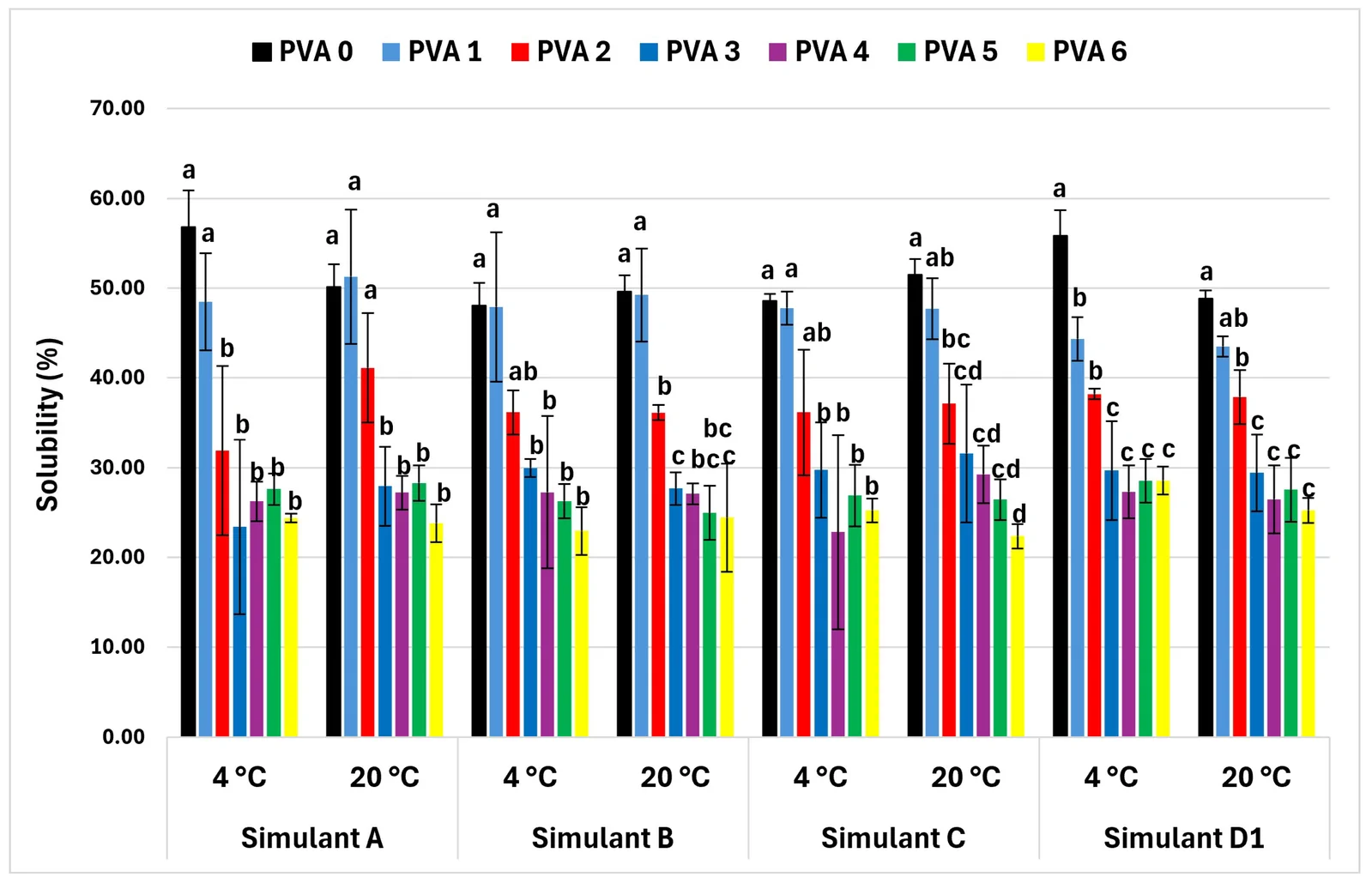
Solubility in food simulants of films PVA 0 (0 wt.% BW), PVA 1 (3.5 wt.% BW), PVA 2 (6.75 wt.% BW), PVA 3 (6.75 wt.% BW), PVA 4 (6.75 wt.% BW), PVA 5 (6.75 wt.% BW), and PVA 6 (6.75 wt.% BW) (simulant A—ethanol 10 wt.%, simulant B—acetic acid 3 wt.%, simulant C—ethanol 20 wt.%, simulants D1—ethanol 50 wt.%) (the same letter means no difference in statistical significance between the values; different letters means that the difference between values is statistically significant (*p* > 0.05, according to one-way ANOVA and the Tukey model)).

All BW-modified films were less soluble in the food simulants tested than the reference film (Figure 6 and Table 9). The lowest solubility values in all the simulants tested were obtained by PVA 3, PVA 4, PVA 5, and PVA 6 films, at both 4 °C and 22 °C (Figure 6). By adding BW in a mass ratio of 12.63–17.79 wt.%, the solubility of the film in all four tested simulants decreased by about 50%. Low values of film solubility indicated high resistance to the tested simulants in [40]; they developed agar/maltodextrin films with BW and found that the solubility of the films obtained decreases in direct proportion to the mass of BW added.

The trend recorded when the solubility is analyzed can be observed also in the case of migration. The migration of the material components into the food simulants was determined as the mass (mg) of migrated material from a surface of 1 dm^2^ in 24 h. Migration drops with the BW mass (Table 9). According to EU Legislation no. 10/2011 (Plastic materials in contact with foodstuffs), the total amount of substances that can migrate from the package should be ≤10 mg/dm^2^ surface or ≤60 mg/kg foodstuff (for large packages). None of the tested materials lies within the requested limit. Moreover, the migration values are considerably higher in all cases, leading to the conclusion that the materials are appropriate only for dry foodstuff.

### 3.11. Water Absorption (Equilibrium Water Content—EWC)

**Figure 7:**
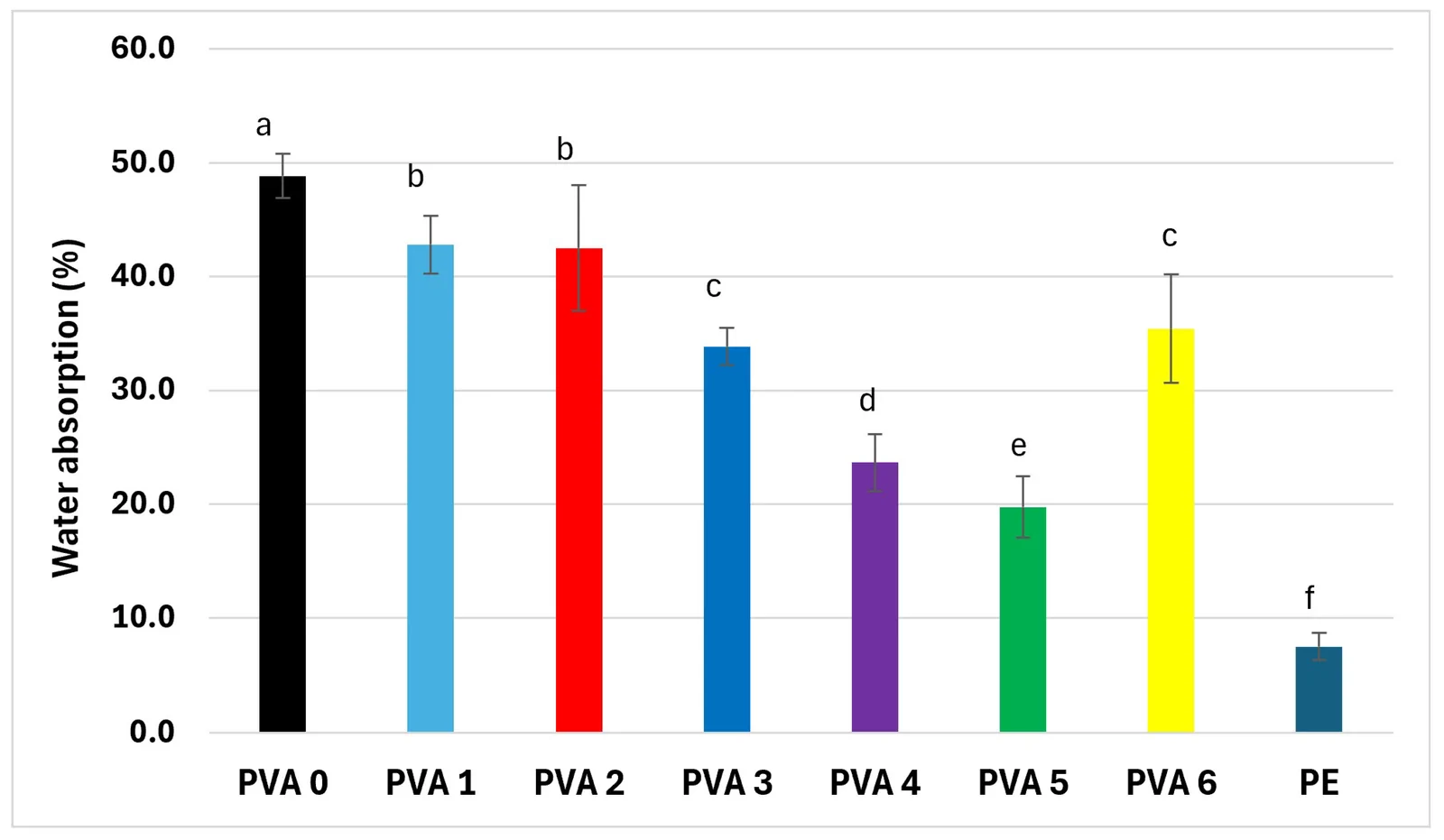
Water absorption on the films PVA 0 (0 wt.% BW), PVA 1 (3.5 wt.% BW), PVA 2 (6.75 wt.% BW), PVA 3 (6.75 wt.% BW), PVA 4 (6.75 wt.% BW), PVA 5 (6.75 wt.% BW), and PVA 6 (6.75 wt.% BW) (PE—polyethylene) (the same letter means no difference in statistical significance between the values; different letters means that the difference between values is statistically significant (*p* > 0.05, according to one-way ANOVA and the Tukey model)).

The water resistance of the materials is an important standard in evaluating their performance as food packages. The water absorption values of the prepared films are illustrated in Figure 7. All PVA films modified with BW presented lower water absorption values compared to the reference film (PVA 0). There were no differences in statistical significance (*p* > 0.05) between PVA 1 and PVA 2 and between PVA 3 and PVA 6. The lowest water absorption capacity was attributed to PVA 4 and PVA 5 samples, which directly correlates with the optimal moisture barrier properties determined for these films (Table 8). PVA 6 showed a higher water absorption capacity than PVA 4 and 5, due to the phase separation between PVA and BW, confirming the morpho-structural analysis. The water absorption capacity decreases by about 50% by adding BW in the proportion of 12.63–15.29 wt.% and is three times higher than that of PE. Farrahnoor et al. [42], who prepared a mycelium-based composite coated with BW and coconut oil in different mass proportions, in order to confer hydrophobicity, demonstrated that water absorption decreases as the amount of wax increases, due to its water-repellent characteristics.

### 3.12. Tensile Strength

**Figure 8:**
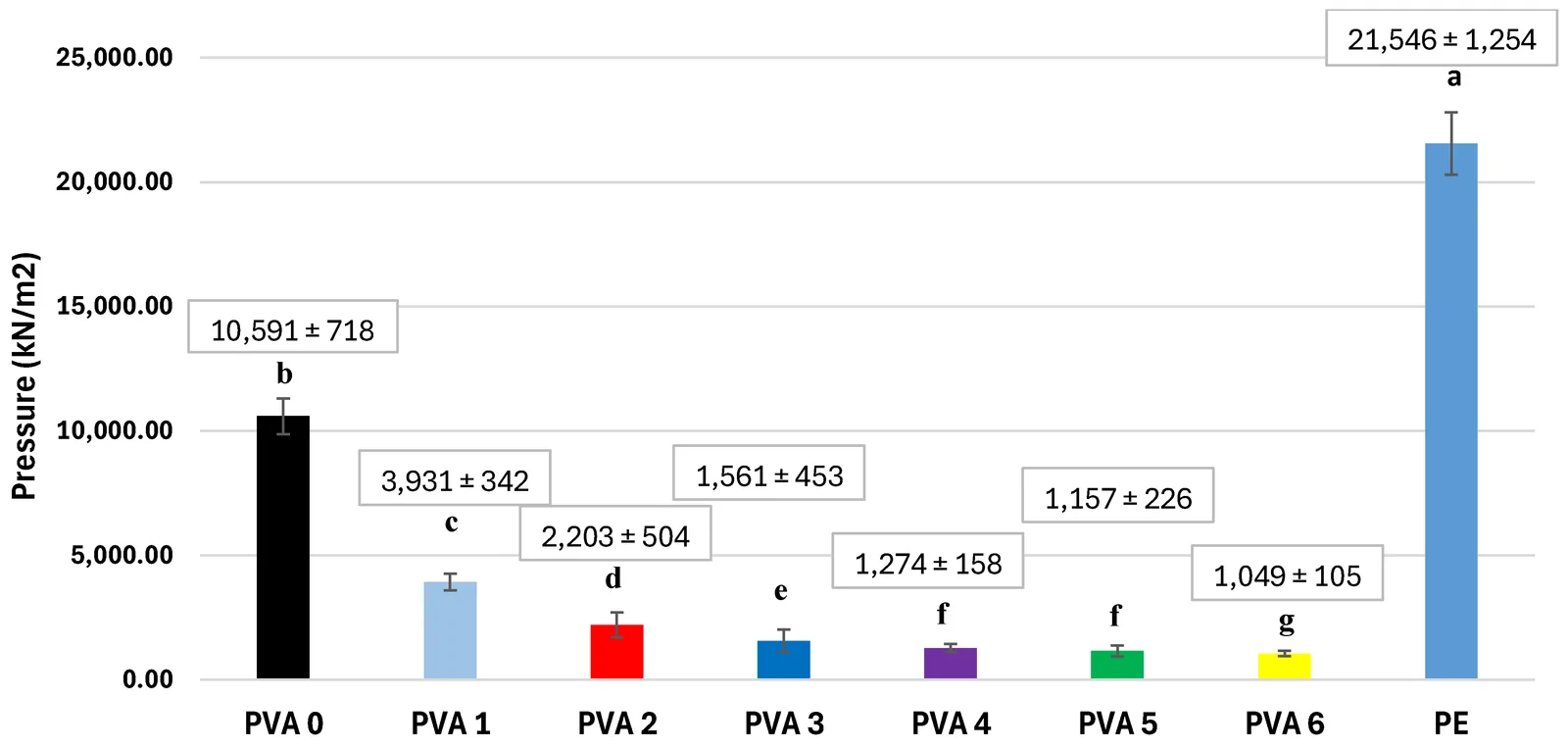
Tensile strength of the films PVA 0 (0 wt.% BW), PVA 1 (3.5 wt.% BW), PVA 2 (6.75 wt.% BW), PVA 3 (6.75 wt.% BW), PVA 4 (6.75 wt.% BW), PVA 5 (6.75 wt.% BW), and PVA 6 (6.75 wt.% BW) (PE—polyethylene) (the same letter means no difference in statistical significance between the values; different letters means that the difference between values is statistically significant (*p* > 0.05, according to one-way ANOVA and the Tukey model)).

Tear strength is a critical quality characteristic of a packaging film, as the integrity of the material can protect food from physical damage and possible contamination from the external environment. The tear strength values obtained for the prepared films are shown in Figure 8. The incorporation of BW into the PVA matrix systematically reduced the tensile strength of the composite films. All wax-containing formulations exhibited values significantly lower than that of the neat PVA control (*p* < 0.05). By adding BW in the proportion of 12.63–17.79 wt.%, the mechanical strength was reduced by 16 times compared to PE and 8 times compared to unchanged PVA. Similar findings were reported by Ariati et al. [20], who prepared polydimethylsiloxane films with the addition of BW in different mass proportions and showed that the tensile strength of BW-modified films decreases with the addition of wax to the film matrix, with linear regression, except for the film containing 4% wax. Lim et al. [43] developed composite materials based on PVA and polyacrylic acid (PAA) modified with BW and they also showed that incorporation of the BW into polymer matrix leads to the significant reduction in the tensile strength, due to the substitution of strong polymer–polymer interactions with weaker polymer–wax interactions. They also established that the drop is pronounced when incorporating BW exceeding 10%, as a result of the inability of excessive beeswax to uniformly disperse within the polymer matrix. Moreover, the BW agglomeration on the surface further reduced the film’s tensile strength. The results showed that the materials are not suitable for use as shopping bags, but for the packaging of food on the shelf.

Improving the mechanical and tear resistance of polyvinyl alcohol (PVA) films modified with BW requires overcoming the inherent phase separation and interfacial incompatibility between the hydrophilic polymer matrix and the hydrophobic wax. To address this, several strategies can be implemented, including the incorporation of reinforcing nanofillers (such as cellulose nanocrystals) [1,3] to form a dense internal hydrogen-bonded network, and chemical crosslinking using agents like citric acid to restrict excessive polymer chain mobility. Additionally, employing effective surfactants or compatibilizers enhances interfacial adhesion and reduces droplet size, which, combined with advanced homogenization techniques such as ultrasonication and optimized wax concentrations, prevents premature structural failure and significantly enhances overall film integrity [8].

## 4. Conclusions

The aim of the study was to establish the optimal amount of BW to be added to PVA in order to obtain the maximum barrier effect against water vapor. In this regard, PVA-based films modified with beeswax ranging from 3.5 wt.% to 17.79 wt.% were developed. From the morphological point of view (SEM and AFM), the presence of BW gives a granular appearance to the surface, the roughness increasing with the mass of BW, and the sample with the highest BW content (17.79 wt.%) showed wax agglomerations on the surface. The degree of crystallization increases with BW from 0 to 9.79 wt.%, showing that this amount of BW does not induce a significant disorganization of the PVA network. In contrast, the crystallization of the film containing 12.63–15.29 wt.% BW decreases compared to the films where BW content was between 3.5 wt.% and 9.79 wt.%, because BW has altered the lattice. A content higher than 15.29 wt.% BW in the film determines the separation of the PVA and BW phases; thus, the degree of crystallization remains comparable to that of the films where BW content was between 3.5 wt.% and 9.79 wt.%. These results are also confirmed by explaining the variations in the preponderance of inter- and intramolecular hydrogen bonds and the free OH groups in the PVA polymer lattice, determined by the deconvolution of the FTIR spectra. Thermogravimetric analyses show that the BW incorporation increased the high-temperature degradation pathway of PVA, without compromising the overall thermal stability of the films. If 12.63–15.29 wt.% BW is added to the film, the WVTR will decrease by 24 times in comparison to the unchanged PVA and only twice that of PE. The WVP varied similarly to the WVTR and decreased with BW up to a BW content of 15.29 wt.%. The solubility and the migration in the four types of food simulants is approximately 50% lower with the addition of 12.63–17.79 wt.% BW. The films’ tensile strength decreases with the addition of 12.63–17.79 wt.% BW, which is 16 times higher than that of PE and 8 times higher than that of unchanged PVA. This implies that the films are exclusively suitable for food packaging on the shelf. These findings are encouraging and indicate that further optimization of the PVA/BW formulation could lead to materials with physicochemical properties comparable to those of petroleum-based food packaging materials.

## Figures and Tables

**Table 1:** The errors showing the films’ homogeneity and the semi-quantitative analysis of the PVA-based films.

FILM (BW Content wt.%)	Error (%) Showing the Films’ Homogeneity	Carbon	Oxygen	Nitrogen	Sodium	Chloride
		(Atom %)				
		From Bulk (EDXS)/from Surface (XPS) *				
PVA 0 (0)	0.6	60 ± 2.5/71.3 ± 2.0 ^e^	37 ± 2.1/24.8 ± 2.5 ^b^	1.0 ± 0.01/3.9 ± 0.4 ^b^	0.9 ± 0.02/0 ^b^	0.9 ± 1.0/0 ^c^
PVA 1 (3.5)	2.5	61 ± 1.1/72.7 ± 2.4 ^e^	34 ± 0.5/23.5 ± 0.4 ^c^	1.3 ± 0.1/3.8 ± 0.1 ^a^	1.3 ± 0.01/0 ^a^	2.5 ± 0.8/0 ^a^
PVA 2 (6.75)	0.3	62 ± 1.5/75.7 ± 2.6 ^e^	33 ± 1.4/21.2 ± 0.5 ^c^	1.2 ± 0.2/3.1 ± 0.2 ^a^	1.2 ± 0.14/0 ^a^	2.8 ± 0.8/0 ^a^
PVA 3 (9.79)	0.7	64 ± 2.2/90.2 ± 2.7 ^d^	33 ± 2.4/8.6 ± 1.4 ^c^	0.5 ± 0.04/1.2 ± 0.1 ^c^	1.2 ± 0.04/0 ^a^	1.7 ± 0.04/0 ^b^
PVA 4 (12.63)	4.1	69 ± 2.1/90.1 ± 2.4 ^c^	28 ± 0.5/8.9 ± 0.8 ^d^	0.5 ± 0.08/1 ± 0.2 ^c^	0.9 ± 0.04/0 ^b^	1.5 ± 0.02/0 ^b^
PVA 5 (15.29)	2.4	70 ± 2.9/91.5 ± 4.5 ^c^	26 ± 1.4/7 ± 1.2 ^d^	0.3 ± 0.04/1.5 ± 0.4 ^d^	1.3 ± 0.2/0 ^a^	2.0 ± 0.1/0 ^b^
PVA 6 (17.79)	0.7	77 ± 2.5/93 ± 3.5 ^b^	21 ± 0.7/5.7 ± 4.2 ^e^	0.3 ± 0.01/1.3 ± 0.5 ^d^	0.7 ± 0.03/0 ^c^	1.0 ± 0.002/0 ^c^
Neat BW	-	93/94 ^a^ [23]	7/6 ^a^ [23]	-	-	-

* The same letter in the same column means no difference in statistical significance between the values; different letters in the same column means that the difference between values is statistically significant (*p* > 0.05, according to one-way ANOVA and the Tukey model).

**Table 2:** Crystallinity and crystallite mean size of the samples determined from XRD.

FILM (BW Content wt.%)	PVA 0 (0)	PVA 1 (3.5)	PVA 2 (6.75)	PVA 3 (9.79)	PVA 4 (12.63)	PVA 5 (15.29)	PVA 6 (17.79)
Crystallinity (%) *	0.52 ± 0.02 ^d^	13.98 ± 0.08 ^c^	26.24 ± 0.95 ^a^	24.08 ± 1.12 ^a^	21.73 ± 0.85 ^b^	20.32 ± 0.65 ^b^	23.31 ± 0.96 ^a^
Crystallite mean size (nm) *	-	33.40 ± 1.45 ^a^	13.16 ± 0.10 ^c^	24.84 ± 0.95 ^b^	24.94 ± 0.75 ^b^	24.76 ± 1.01 ^b^	21.80 ± 0.74 ^b^

* The same letter in the same line means no difference in statistical significance between the values; different letters in the same line means that the difference between values is statistically significant (*p* > 0.05, according to one-way ANOVA and the Tukey model).

**Table 3:** Preponderance of free -OH and intra- and intermolecular -H bonds (R—correlation coefficient).

FILM (BW Content wt.%)	Free OH (%) *	R^2^	Intramolecular H Bond (%) *	R^2^	Intermolecular H Bond (%) *	R^2^
PVA 0 (0)	0.0016 ± 0.0001 ^e^	0.998	13.27 ± 0.75 ^e^	0.994	86.72 ± 0.79 ^a^	0.998
PVA 1 (3.5)	5.26 ± 0.17 ^d^	0.997	25.45 ± 0.24 ^d^	0.995	69.29 ± 0.88 ^b^	0.997
PVA 2 (6.75)	7.56 ± 0.23 ^c^	0.987	28.24 ± 0.63 ^c^	0.991	64.2 ± 0.42 ^b^	0.992
PVA 3 (9.79)	10.35 ± 0.12 ^b^	0.992	32.45 ± 1.45 ^b^	0.998	57.2 ± 1.42 ^c^	0.998
PVA 4 (12.63)	11.25 ± 0.45 ^b^	0.991	35.45 ± 1.77 ^b^	0.992	53.3 ± 1.01 ^c^	0.991
PVA 5 (15.29)	15.24 ± 0.75 ^a^	0.994	41.25 ± 1.54 ^a^	0.993	43.51 ± 2.45 ^d^	0.995
PVA 6 (17.79)	4.25 ± 0.14 ^d^	0.990	27.18 ± 0.85 ^c^	0.987	68.57 ± 3.47 ^b^	0.994

* The same letter in the same column means no difference in statistical significance between the values; different letters in the same column means that the difference between values is statistically significant (*p* > 0.05, according to one-way ANOVA and the Tukey model).

**Table 4:** Deconvoluted C1s data of the films.

FILM (BW Content wt.%)	Interconnection C-C Between PVA and BW (eV)	% at	C-C/ C-H (eV)	% at	C-OH/ C-O-C (eV)	% at	C-N/C-N^+^ (eV)	% at	C=O/ O-C=O (Ester) −COO Carboxilic (eV)	% at
PVA 0 (0)	absent	-	284.6	54.6	286	24.4	287.4	5.8	288.8	15.2
PVA 1 (3.5)	absent	-	284.6	56.7	286	23.1	287.4	5.6	288.8	14.6
PVA 2 (6.75)	absent	-	284.6	61.5	286.1	22	287.5	4.9	288.9	11.6
PVA 3 (9.79)	283.4	3.7	284.6	81.9	286.1	8.1	287.4	2.2	288.9	4.1
PVA 4 (12.63)	283.4	0.8	284.6	83.9	286	7.3	287	3.1	288.9	4.9
PVA 5 (15.29)	283.4	5.5	284.6	79	286	12.1	287.3	0.7	288.3	2.7
PVA 6 (17.79)	283.3	0.8	284.6	89.8	286.1	5.8	287.3	0.5	288.7	3.2

**Table 5:** Deconvoluted O1s and N1s data of the obtained films.

FILM (BW Content wt.%)	O1s				N1s			
	O(C=O)-C (eV)	% at	C-OH/C-O-C O-(C=O)-C (eV)	% at	C-N (eV)	% at	–C-N^+^ (eV)	% at
PVA 0 (0)	531.8	60.9	533	39.1	399.6	49.8	402.1	50.2
PVA 1 (3.5)	531.8	60.3	533	39.7	399.6	49.4	402.1	50.6
PVA 2 (6.75)	531.9	64.4	533.1	35.6	399.6	47.6	402.1	52.4
PVA 3 (9.79)	531.9	50.5	533.1	49.5	399.7	55.3	402.1	44.7
PVA 4 (12.63)	531.9	57	533.1	43	399.6	52.7	402.2	47.3
PVA 5 (15.29)	531.4	36.6	532.6	63.4	399.7	52.2	402.3	47.8
PVA 6 (17.79)	531.8	55.9	533.1	44.1	-	-	-	-

**Table 6:** AFM data extracted from the topographic images.

FILM (BW Content wt.%)	Medium Roughness (nm) *		Average Quadratic Roughness (nm) *		Average Particle Size (nm) *	
	1 × 1 µm^2^	10 × 10 µm^2^	1 × 1 µm^2^	10 × 10 µm^2^	1 × 1 µm^2^	10 × 10 µm^2^
PVA 0 (0)	0.29 ± 0.01 ^d^	5.57 ± 0.9 ^e^	0.36 ± 0.02 ^d^	7.18 ± 0.8 ^f^	Flat surface	Flat surface
PVA 1 (3.5)	0.32 ± 0.05 ^d^	15.83 ± 1.8 ^d^	0.44 ± 0.04 ^d^	23.73 ± 1.5 ^e^	11.77 ± 1.7 ^d^	Flat surface
PVA 2 (6.75)	0.57 ± 0.04 ^c^	60.51 ± 2.5 ^c^	0.75 ± 0.05 ^c^	91.40 ± 5.7 ^c^	24.45 ± 2.5 ^C^	667.11 ± 15.8 ^c^
PVA 3 (9.79)	0.64 ± 0.02 ^c^	53.41 ± 2.4 ^c^	0.82 ± 0.01 ^c^	75.68 ± 6.5 ^d^	20.61 ± 3.4 ^c^	510.02 ± 20.4 ^d^
PVA 4 (12.63)	0.90 ± 0.04 ^b^	98.20 ± 3.5 ^b^	1.22 ± 0.98 ^b^	128.7 ± 14.5 ^b^	30.11 ± 2.8 ^b^	670.14 ± 2.14 ^c^
PVA 5 (15.29)	1.09 ± 0.08 ^a^	119.63 ± 10.5 ^a^	1.58 ± 0.45 ^a^	148.82 ± 10.2 ^a^	34.92 ± 2.9 ^b^	784.10 ± 14.7 ^b^
PVA 6 (17.79)	1.17 ± 0.12 ^a^	130.96 ± 14.5 ^a^	1.87 ± 0.8 ^a^	164.38 ± 14.7 ^a^	41.88 ± 4.7 ^a^	988.98 ± 24.5 ^a^

* The same letter in the same column means no difference in statistical significance between the values; different letters in the same column means that the difference between values is statistically significant (*p* > 0.05, according to one-way ANOVA and the Tukey model).

**Table 7:** DTA and TGA parameters of the films.

FILM (BW Content wt.%)	DTA Events	TG Mass Loss	Total Mass Loss
PVA 0 (0)	Endo 107 °C melting of PVA and vaporization of Gly Endo 391 °C, 431 °C, 498 °C—polymer scission and decomposition	107 °C: −11.7% 150–250 °C: −35.2% 250–300 °C: −17.4% 300–400 °C: −20.2% 400–500 °C: −14.1%	−98.6%
PVA 1 (3.5)	Endo 77 °C melting of BW Endo 145 °C vaporization and degradation of Gly Endo 404 °C, 493 °C polymer scission and decomposition	77 °C: −7.1% 150–250 °C: −29.7% 250–300 °C: −17.5% 300–400 °C: −27.1% 400–500 °C: −17.8%	−99.2%
PVA 2 (6.75)	Endo 79 °C melting of BW Endo 150 °C vaporization and degradation of Gly Endo 439 °C, 573 °C polymer scission and decomposition	79 °C: −4.9% 150–250 °C: −22.6% 250–300 °C: −19.7% 300–400 °C: −30.6% 400–500 °C: −21.5%	−99.3%
PVA 3 (9.79)	Endo 78 °C melting of BW Endo 142 °C vaporization and degradation of Gly Endo 350 °C, 429 °C, 487 °C polymer scission and decomposition	78 °C: −2.7% 150–250 °C: −18.4% 250–300 °C: −16.7% 300–400 °C: −36.9% 400–500 °C: −24.7%	−99.4%
PVA 4 (12.63)	Endo 75 °C melting of BW Endo 170 °C vaporization and degradation of Gly Endo 340 °C, 432 °C, 483 °C polymer scission and decomposition	75 °C: −4% 150–250 °C: −17.7% 250–300 °C: −17% 300–400 °C: −40.8% 400–500 °C: −19.9%	−99.4%
PVA 5 (15.29)	Endo 79 °C melting of BW Endo 172 °C vaporization and degradation of Gly Endo 478 °C, 563 °C polymer scission and decomposition	79 °C: −3.9% 150–250 °C: −8.3% 250–300 °C: −17% 300–400 °C: −41.8% 400–600 °C: −28.7%	−99.7%
PVA 6 (17.79)	Endo 85 °C melting of BW Endo 175 °C vaporization and degradation of Gly Endo 425 °C, 478 °C polymer scission and decomposition	85 °C: −1.7% 150–250 °C: −15.5% 250–300 °C: −15.9% 300–400 °C: −41.2% 400–500 °C: −26.3%	−99.6%

**Table 8:** Water vapor transmission rate (WVTR) and water vapor permeability (WVP) values of the films (PE—polyethylene).

FILM (BW Content wt.%)	WVTR (g/m^2^ · 24 h) *	Thickness (mm)	WVP × 10^10^ (g/s m Pa)
PE	50.3 ± 1.5 (d)	0.01 ± 0.0001 (e)	0.2 ± 0.02 (e)
PVA 0 (0)	2035.3 ± 18.3 (a)	0.236 ± 0.005 (d)	3.11 ± 0.316 (b)
PVA 1 (3.5)	412.4 ± 30.5 (b)	0.479 ± 0.014 (c)	3.24 ± 0.465 (a)
PVA 2 (6.75)	409.4 ± 45.8 (b)	0.453 ± 0.025 (c)	2.67 ± 0.587 (b)
PVA 3 (9.79)	357 ± 2.8 (b)	0.648 ± 0.093 (b)	3.28 ± 0.471 (a)
PVA 4 (12.63)	103.6 ± 17.7 (d)	0.721 ± 0.039 (a)	2.99 ± 0.35 (b)
PVA 5 (15.29)	**82.5 ± 7.7 (d)**	0.661 ± 0.089 (b)	**2.41 ± 0.34 (d)**
PVA 6 (17.79)	186 ± 26.8 (c)	0.766 ± 0.018 (a)	2.69 ± 0.56 (c)

* The same letter in the same column means no difference in statistical significance between the values; different letters in the same column means that the difference between values is statistically significant (*p* > 0.05, according to one-way ANOVA and the Tukey model).

**Table 9:** Values of migration (mg/dm^2^ in 24 h) of materials into food simulants.

FILM (BW Content wt.%)	Simulant A * (Ethanol 10 wt.%)		Simulant B * (Acetic Acid 3 wt.%)		Simulant C * (Ethanol 20 wt.%)		Simulant D1 * (Ethanol 50 wt.%)	
	20 °C	4 °C	20 °C	4 °C	20 °C	4 °C	20 °C	4 °C
PVA 0 (0)	686.67 ± 42.52 ^a^	470.00 ± 35.36 ^c^	745.00 ± 27.84 ^a^	630.00 ± 21.21 ^a^	425.00 ± 14.14 ^a^	251.67 ± 7.64 ^a^	358.33 ± 17.56 ^b^	310.00 ± 10.00 ^a^
PVA 1 (3.5)	692.50 ± 10.61 ^a^	423.33 ± 22.55 ^d^	782.50 ± 3.54 ^a^	638.33 ± 23.63 ^a^	435.00 ± 21.21 ^a^	265.00 ± 0.00 ^a^	340.00 ± 35.36 ^b^	321.67 ± 7.64 ^a^
PVA 2 (6.75)	665.00 ± 42.43 ^a^	502.50 ± 24.75 ^b^	640.00 ± 49.24 ^b^	620.00 ± 28.28 ^a^	372.50 ± 3.54 ^b^	270.00 ± 7.07 ^a^	390.00 ± 7.07 ^a^	325.00 ± 27.84 ^a^
PVA 3 (9.79)	685.00 ± 35.36 ^a^	512.50 ± 10.61 ^b^	559.00 ± 28.16 ^c^	617.50 ± 3.54 ^a^	337.50 ± 38.89 ^b^	206.67 ± 7.64 ^b^	312.50 ± 10.61 ^c^	211.67 ± 10.41 ^b^
PVA 4 (12.63)	527.50 ± 17.68 ^b^	482.50 ± 38.89 ^b^	556.67 ± 35.47 ^c^	635.00 ± 35.00 ^a^	342.50 ± 24.75 ^b^	156.67 ± 15.28 ^c^	358.33 ± 5.77 ^b^	171.67 ± 18.93 ^c^
PVA 5 (15.29)	545.00 ± 70.71 ^b^	547.50 ± 3.54 ^a^	443.33 ± 10.41 ^d^	527.50 ± 17.68 ^b^	333.33 ± 16.07 ^b^	161.67 ± 17.56 ^c^	333.33 ± 15.28 ^b^	152.50 ± 17.68 ^c^
PVA 6 (17.79)	407.50 ± 38.89 ^c^	507.50 ± 31.82 ^b^	441.67 ± 7.64 ^d^	455.00 ± 49.50 ^c^	313.33 ± 22.55 ^c^	130.00 ± 5.00 ^d^	311.67 ± 2.89 ^c^	150.00 ± 10.00 ^c^

* The same letter on the same column means no difference in statistical significance between the values, according to one-way ANOVA and the Tukey model.

## Data Availability

The original contributions presented in this study are included in the article/Supplementary Material. Further inquiries can be directed to the corresponding author.

## Supplementary Materials

The following supporting information can be downloaded at: https://www.mdpi.com/article/10.3390/foods15214006/s1, Figure S1: FTIR spectra of three replicas from each PVA-based film, showing the reproducibility of the preparation route: PVA 0 (0 wt.% BW) (a), PVA 1 (3.5 wt.% BW) (b), PVA 2 (6.75 wt.% BW) (c), PVA 3 (6.75 wt.% BW) (d), PVA 4 (6.75 wt.% BW) (e), PVA 5 (6.75 wt.% BW) (f) and PVA 6 (6.75 wt.% BW) (g); Figure S2. Macroscopic images of the PVA-based films: PVA 0 (0 wt.% BW) (a), PVA 1 (3.5 wt.% BW) (b), PVA 2 (6.75 wt.% BW) (c), PVA 3 (6.75 wt.% BW) (d), PVA 4 (6.75 wt.% BW) (e), PVA 5 (6.75 wt.% BW) (f) and PVA 6 (6.75 wt.% BW) (g); Figure S3. SEM images of the films (a,b) PVA 0 (0 wt.% BW); (c,d) PVA 1 (3.5 wt.% BW); (e,f) PVA 2 (6.75 wt.% BW); (g,h) PVA 3 (6.75 wt.% BW); (i,j) PVA 4 (6.75 wt.% BW); (k,l) PVA 5 (6.75 wt.% BW) and (m,n) PVA 6 (6.75 wt.% BW); Figure S4. Deconvoluted FTIR spectra in the region 3000–3600 cm^−1^ of PVA 0 (0 wt.% BW) (a), PVA 1 (3.5 wt.% BW) (b), PVA 2 (6.75 wt.% BW) (c), PVA 3 (6.75 wt.% BW) (d), PVA 4 (6.75 wt.% BW) (e), PVA 5 (6.75 wt.% BW) (f) and PVA 6 (6.75 wt.% BW) (g); Figure S5. Deconvolution of C1s and O1s XPS spectra of PVA 0–PVA 6 films (PVA 0 (0 wt.% BW), PVA 1 (3.5 wt.% BW), PVA 2 (6.75 wt.% BW), PVA 3 (6.75 wt.% BW), PVA 4 (6.75 wt.% BW), PVA 5 (6.75 wt.% BW) and PVA 6 (6.75 wt.% BW)).

## References

[1] Florido-Moreno P., Benítez J.J., González-Buesa J., Porras-Vázquez J.M., Hierrezuelo J., Grifé-Ruiz M., Romero D., Athanassiou A., Heredia-Guerrero J.A., Guzmán-Puyol S. (2024). Plasticized cellulose bioplastics with beeswax for the fabrication of multifunctional, biodegradable active food packaging. Food Hydrocoll..

[2] Brito-Pereira R., Ribeiro C., Tubio C.R., Castro N., Costa P., Lanceros-Mendez S. (2022). Beeswax multifunctional composites with thermal-healing capability and recyclability. Chem. Eng. J..

[3] Trinh B.M., Chang B.P., Mekonnen T.H. (2023). The barrier properties of sustainable multiphase and multicomponent packaging materials: A review. Prog. Mater. Sci..

[4] Adelnia H., Ensandoost R., Moonshi S.S., Gavgani J.N., Vasafi E.I., Ta H.T. (2022). Freeze/thawed polyvinyl alcohol hydrogels: Present, past and future. Eur. Polym. J..

[5] Deng H., Su J., Zhang W., Khan A., Goksen G., Kashyap P., Ezati P., Rhim J.W. (2024). A review of starch/polyvinyl alcohol (PVA) blend film: A potential replacement for traditional plastic-based food packaging film. Int. J. Biol. Macromol..

[6] Al-Tayyar N.A., Youssef A.M., Al-hindi R. (2020). Antimicrobial food packaging based on sustainable Bio-based materials for reducing foodborne Pathogens: A review. Food Chem..

[7] Cheng Y., Zhai X., Wu Y., Li C., Zhang R., Sun C., Wang W., Hou H. (2023). Effects of natural wax types on the physicochemical properties of starch/gelatin edible films fabricated by extrusion blowing. Food Chem..

[8] Mathew A., Poulose A., Peter A., Lal H.M., Uthaman A., Saheed M.S., Pasquini D., Grohens Y., Gopakumar D.A., George J.J. (2025). Bio-inspired hydrophobicity in cellulose nanopaper via thermal-induced phase separation of beeswax: A new strategy to develop sustainable food packaging materials and its service life prediction. Food Packag. Shelf Life.

[9] Bellelli M., Licciardello F., Pulvirenti A., Fava P. (2018). Properties of poly(vinyl alcohol) films as determined by thermal curing and addition of polyfunctional organic acids. Food Packag. Shelf Life.

[10] Alshehri A.A., Hamed Y.S., Kamel R.M., Shawir S.M.S., Sakr H., Ali M., Ammar A., Saleh M.N., Fadly E.E., Salama M.A. (2024). Enhanced physical properties, antioxidant and antibacterial activity of bio-composite films composed from carboxymethyl cellulose and polyvinyl alcohol incorporated with broccoli sprout seed extract for butter packaging. Int. J. Biol. Macromol..

[11] Koosha M., Hamedi S. (2019). Intelligent Chitosan/PVA nanocomposite films containing black carrot anthocyanin and bentonite nanoclays with improved mechanical, thermal and antibacterial properties. Prog. Org. Coat..

[12] Oun A.A., Shin G.H., Kim J.T. (2022). Antimicrobial; antioxidant, and pH-sensitive polyvinyl alcohol/chitosan-based composite films with aronia extract, cellulose nanocrystals, and grapefruit seed extract. Int. J. Biol. Macromolec..

[13] Liu Y., Shi D., Fan X., Yin C., Huang Z., Meng J., Deng B., Liu Y., Chen C., Gao H. (2025). Preparation and characterization of a novel polyvinyl alcohol/pectin film containing bromocresol green-alizarin mixed indicator label for monitoring mushroom freshness. Food Packag. Shelf Life.

[14] Dudeja I., Mankoo R.K., Singh A., Kaur J. (2023). Citric aid: An ecofriendly cross-linker for the production of functional biopolymeric materials. Sustain. Chem. Pharm..

[15] Lai D.S., Osman A.F., Adnan S., Ibrahim I., Nabil A., Mustapha M. (2023). Thermoplastic starch hybrid biocomposite films with improved strength and flexibility produced through crosslinking via carboxylic acid. J. Thermoplast. Compos. Mat..

[16] Zhang R., Zhai X., Wang W., Hou H. (2022). Preparation and evaluation of agar/maltodextrin-beeswax emulsion films with various hydrophilic-lipophilic balance emulsifiers. Food Chem..

[17] Qazanfarzadeh Z., Kumaravel V. (2023). Hydrophobisation approaches of protein-based bioplastics. Trends Food Sci. Technol..

[18] Hosseini S.F., Mousavi Z., McClements D.J. (2023). Beeswax: A review on the recent progress in the development of superhydrophobic films/coatings and their applications in fruits preservation. Food Chem..

[19] Aggarwal S., Kathuria D., Singh N. (2025). Edible films and coatings, its chemical crosslinking, starch-protein interaction and application in food system: A systematic review. Int. J. Biol. Macromol..

[20] Ariati R., Souza A., Souza M., Zille A., Soares D., Lima R., Ribeiro J. (2024). Mechanical and optical properties assessment of an innovative PDMS/Beeswax composite for a wide range of applications. J. Mech. Behav. Biomed. Mater..

[21] Ahn K., Kambiz S., Park K., Seo J. (2024). Enhancing polyvinyl alcohol surface properties through pre-drying treatment and modified electrospun polycaprolactone nanofibers. Prog. Org. Coat..

[22] Chen N., Zhang J. (2010). The role of hydrogen-bonding interaction in poly(vinylalcohol)/poly(acrylic acid) blending solutions and their films. Chin. J. Polym. Sci..

[23] Kameda T. (2004). Molecular structure of crude beeswax studied by solid-state C13 NMR. J. Insect Sci..

[24] Hromiš N.M., Lazić V.L., Markov S.L., Popović S.Z., Šuput D.Z., Džinić N.R., Velićanski A.S., Popović L.M. (2015). Optimization of chitosan biofilm properties by addition of caraway essential oil and beeswax. J. Food Eng..

[25] Qamar S.A., Piccolella S., Ziegler G.R., Pacifico S., Zhang Y. (2025). Functional bioplastic films from cocoa shell cellulose and natural waxes: Toward sustainable active packaging. Food Packag. Shelf Life.

[26] Zhang R., Wang W., Zhang H., Dai Y., Dong H., Kong L., Hou H. (2020). Effects of preparation conditions on the properties of agar/maltodextrin-beeswax pseudo-bilayer films. Carbohydr. Polym..

[27] Peter A., Pop L., Cozmuta L.M., Nicula C., Cozmuta A.M., Drazic G., Magyari K., Muresan-Pop M., Todea M., Baia L. (2024). Beeswax-poly(vinyl alcohol) composite films for bread packaging. Food Chem. X.

[28] Huang H., Huang C., Xu C., Liu R. (2022). Development and characterization of lotus-leaf-inspired bionic antibacterial adhesion film through beeswax. Food Packag. Shelf Life.

[29] Diyana Z.N., Jumaidin R., Selamat M.Z., Suan M.S.M. (2021). Thermoplastic starch/beeswax blend: Characterization on thermal mechanical and moisture absorption properties. Int. J. Biol. Macromol..

[30] Ponnusamy P., Loganathan M., Krishnasamy S. (2025). Mass transfer modelling and nitrogen release kinetics of nano-biochar dispersed PVA/PVP matrix encapsulated slow-release fertilizer for sustainable agriculture. Chem. Eng. J. Adv..

[31] Mabied A.F., Tiama T.M., El-Sayed A.F., Ibrahim M.A. (2025). Structural characterizations, DFT, and docking of novel chitosan/PVA/germanium oxide as a potent antimicrobial agent. Int. J. Biol. Macromol..

[32] Gaillard Y., Mija A., Burr A., Darque-Ceretti E., Felder E., Sbirrazzuoli N. (2011). Green material composites from renewable resources: Polymorphic transitions and phase diagram of beeswax/rosin resin. Thermochim. Acta.

[33] Zhang Z., Fang C., Zhang X., Zhang W., Wang D. (2025). Biodegradable laminated poly(butylene adipate-co-terephthalate) (PBAT)/grasshopper protein-based multilayer films coated with chitosan/beeswax/baicalein for active packaging. Future Foods.

[34] Mo J., Lei J., Wang H., Kang Q., Liu W., Qiu X. (2024). Melt-processable polyvinyl alcohol/lignin composites with improved strength via synergistic plasticization of lignin. Int. J. Biol. Macromol..

[35] Gilormini P., Verdu J. (2018). On the role of hydrogen bondingon water absorption in polymers. Polymer.

[36] Zuber B., Cersoy S., Herbin M., Sablier M., Rouchon V. (2021). Beeswax-rosin mixtures in historical wet collection sealants: Qualitative analysis of their composition by DSC and ATR-FTIR spectroscopy. Vib. Spectrosc..

[37] Briggs A., Seah J.C.R. (1990). Handbook of X-ray Photoelectron Spectroscopy. Proceedings of the First International Symposium on Advanced Materials for ULSI.

[38] Chan C.M., Weng L.-T. (2016). Surface Characterization of Polymer Blends by XPS and ToF-SIMS. Materials.

[39] Almazrouei M., Adeyemi I., Janajreh I. (2022). Thermogravimetric assessment of the thermal degradation during combustion of crude and pure glycerol. Biomass Convers. Biorefinery.

[40] Zhang R., Wang W., Zhang H., Dai Y., Dong H., Hou H. (2019). Effects of hydrophobic agents on the physicochemical properties of edible agar/maltodextrin films. Food Hydrocoll..

[41] Oliveira V.R.L., Santos F.K.G., Leite R.H.L., Silva K.N.O. (2018). Use of biopolymeric coating hydrophobized with beeswax in post-harvest conservation of guavas. Food Chem..

[42] Farrahnoor A., Sazali N.A.A., Yusoff H., Zhou B.T. (2024). Effect of beeswax and coconut oil as natural coating agents on morphological, degradation behaviour, and water barrier properties of mycelium-based composite in modified controlled environment. Prog. Org. Coat..

[43] Lim W.S., Kim M.H.K., Park H.J., Lee M.H. (2024). Characterization of Polyvinyl Alcohol (PVA)/Polyacrylic Acid (PAA) Composite Film-Forming Solutions and Resulting Films as Affected by Beeswax Content. Polymers.

