# Supplementary material for: Influence of Beeswax Content on Barrier Properties of PVA-Based Films
Source: Foods. 2026 Nov 9;15(21):4006. doi: 10.3390/foods15214006 (PMC13763312; doi:10.3390/foods15214006)
Supplement: Supplementary file 1 [file foods-15-04006-s001.zip › foods-4646282-supplementary.pdf]

Influence of beeswax content on PVA-based films

Lucica Maria Pop[a], Camelia Nicula[a], Alexandra Maria Uivarasan[a], Zorica Voşgan[a], Monica Liliana Marian[a], Cristina Mihali[a], Lucia Mihalescu[a], Oana Mare Rosca[a], Monica Tarau[a], Ionela Traistaru Marcu[a], Adrian Matei Cosma[a], Doina Boitor Hontau[a]
Goran Drazic[b], Klara Magyari[c], Marieta Muresan-Pop[c], Ioan Botiz[c d e], Milica Todea[c f], Lucian Baia[c d e] and Anca Peter[a*]

Supplementary material

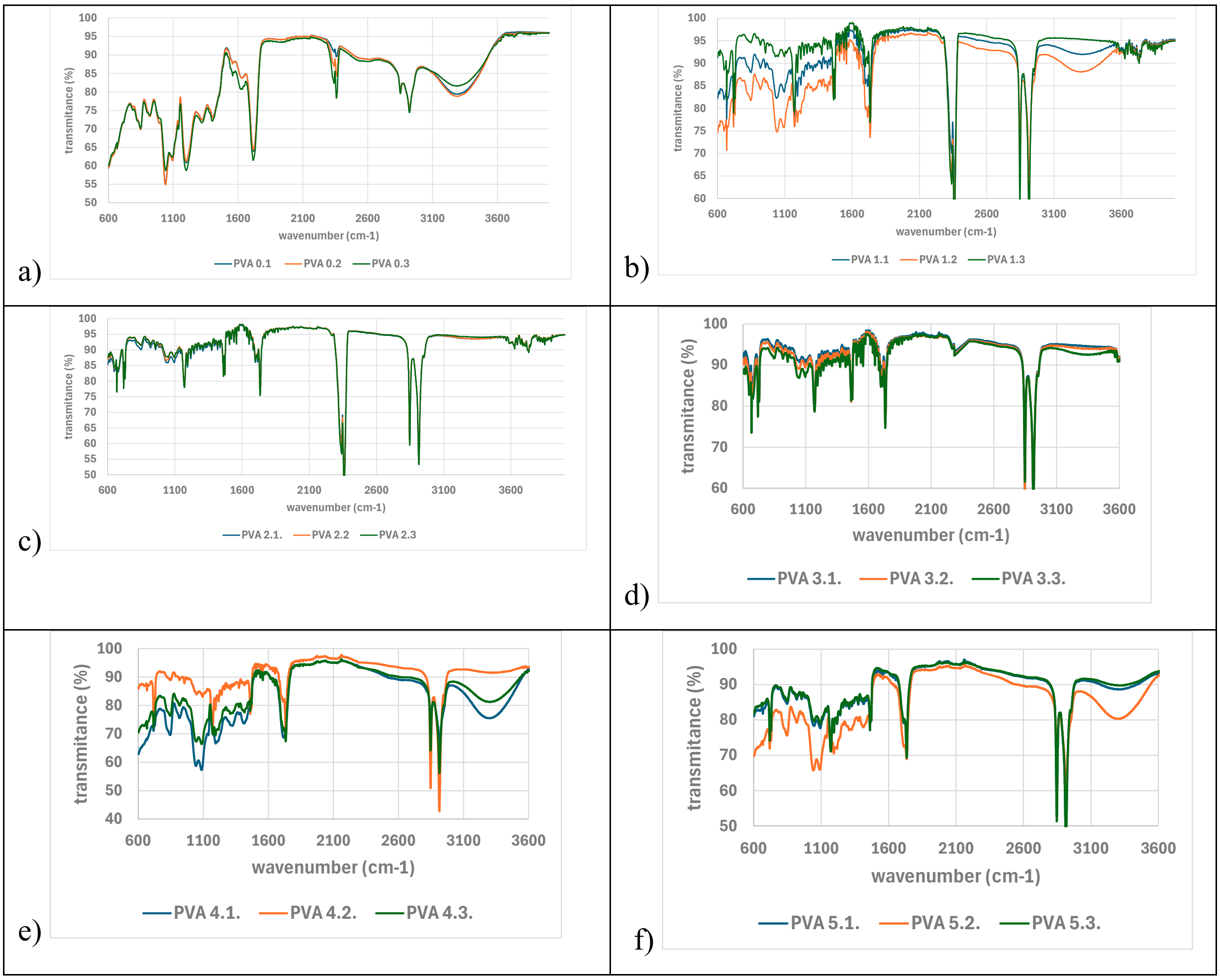

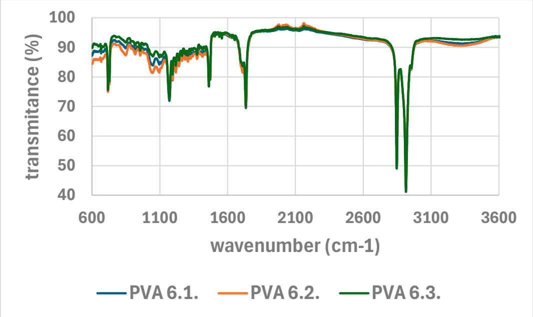

g)

Figure S1. FTIR spectra of three replicas from each PVA-based film, showing the reproducibility of the preparation route: PVA 0 (0 wt.% BW) a), PVA 1 (3.5 wt.% BW) b), PVA 2 (6.75 wt.% BW) c), PVA 3 (6.75 wt.% BW) d), PVA 4 (6.75 wt.% BW) e), PVA 5 (6.75 wt.% BW) f) and PVA 6 (6.75 wt.% BW) g)

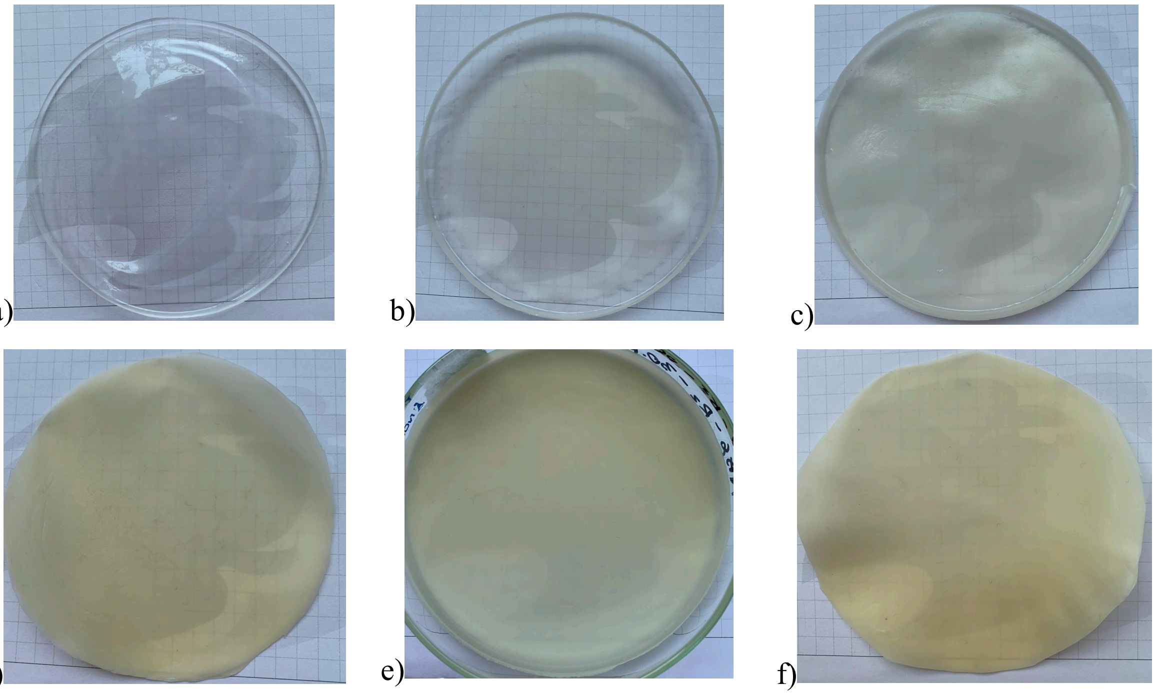

a) b) c) d) e) f)

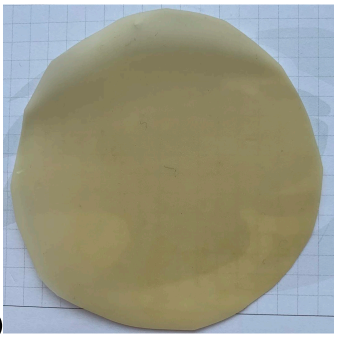

g)

Figure S2. Macroscopic images of the PVA-based films: PVA 0 (0 wt.% BW) a), PVA 1 (3.5 wt.% BW) b), PVA 2 (6.75 wt.% BW) c), PVA 3 (6.75 wt.% BW) d), PVA 4 (6.75 wt.% BW) e), PVA 5 (6.75 wt.% BW) f) and PVA 6 (6.75 wt.% BW) g)

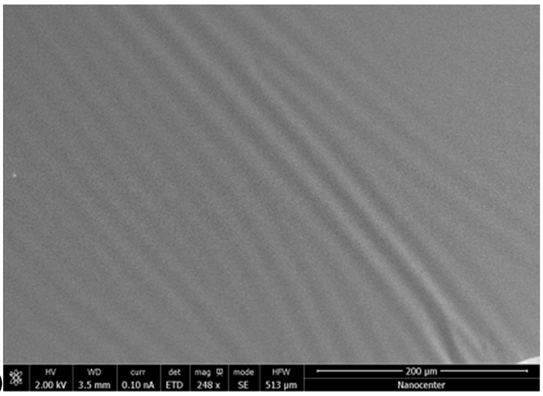
HV 2.00 kV
WD 3.5 mm
curr 0.10 nA
det ETD
mag 248 x
mode SE
HFW 513 µm
200 µm
Nanocenter
a)

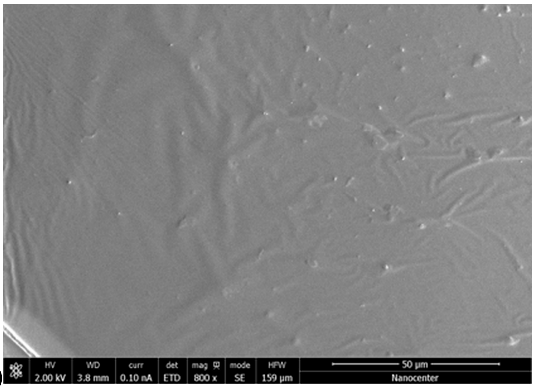
HV 2.00 kV
WD 3.8 mm
curr 0.10 nA
det ETD
mag 800 x
mode SE
HFW 159 µm
50 µm
Nanocenter
b)

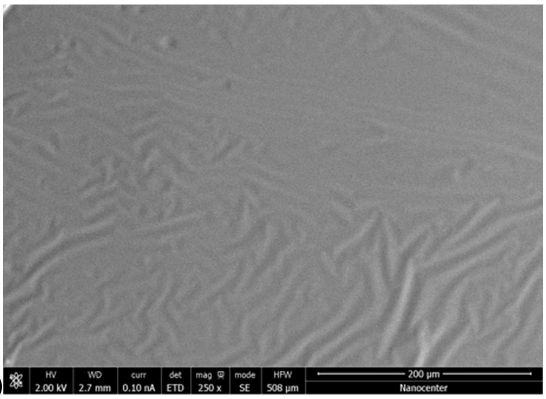
HV 2.00 kV
WD 2.7 mm
curr 0.10 nA
det ETD
mag 250 x
mode SE
HFW 508 µm
200 µm
Nanocenter
c)

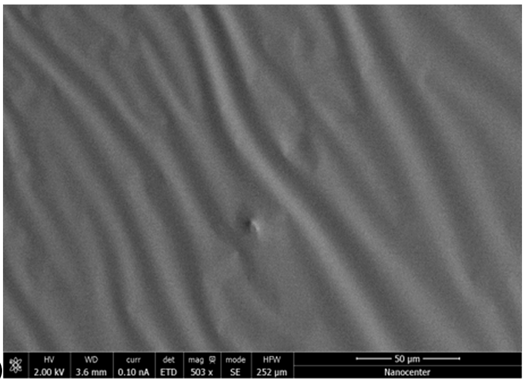
HV 2.00 kV
WD 3.6 mm
curr 0.10 nA
det ETD
mag 503 x
mode SE
HFW 252 µm
50 µm
Nanocenter
d)

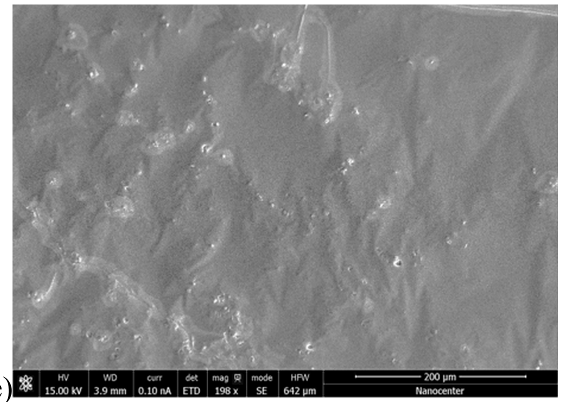
HV 15.00 kV
WD 3.9 mm
curr 0.10 nA
det ETD
mag 198 x
mode SE
HFW 642 µm
200 µm
Nanocenter
e)

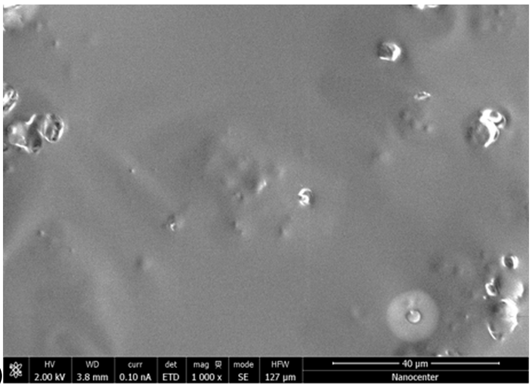
HV 2.00 kV
WD 3.8 mm
curr 0.10 nA
det ETD
mag 1 000 x
mode SE
HFW 127 µm
40 µm
Nanocenter
f)

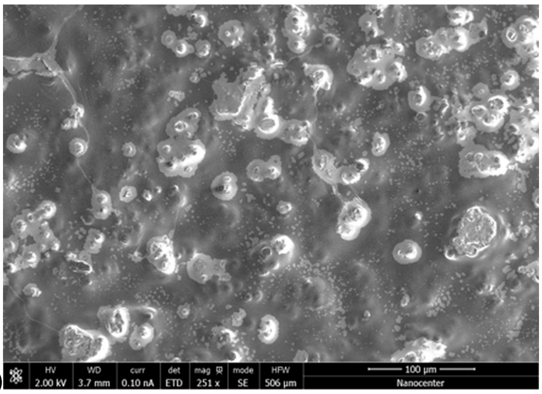
HV WD curr det mag mode HFW
2.00 kV 3.7 mm 0.10 nA ETD 251 x SE 506 µm
100 µm
Nanocenter
g)

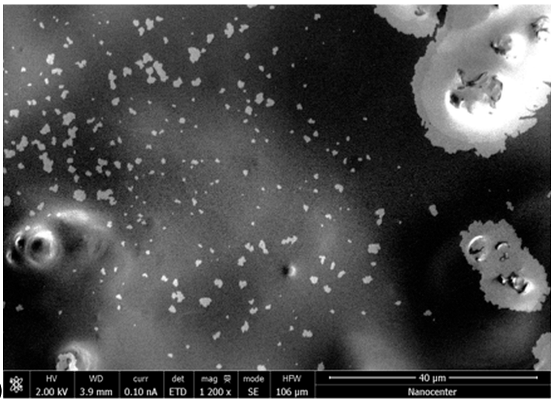
HV WD curr det mag mode HFW
2.00 kV 3.9 mm 0.10 nA ETD 1 200 x SE 106 µm
40 µm
Nanocenter
h)

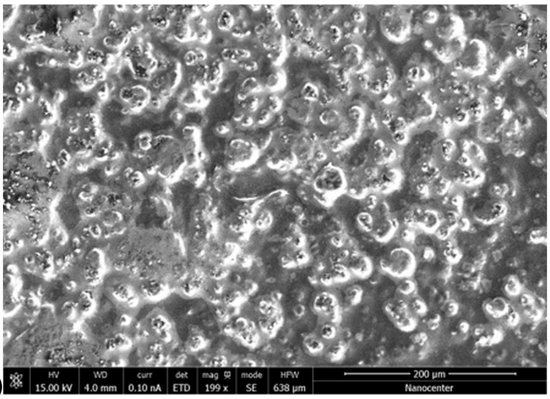
HV WD curr det mag mode HFW
15.00 kV 4.0 mm 0.10 nA ETD 199 x SE 638 µm
200 µm
Nanocenter
i)

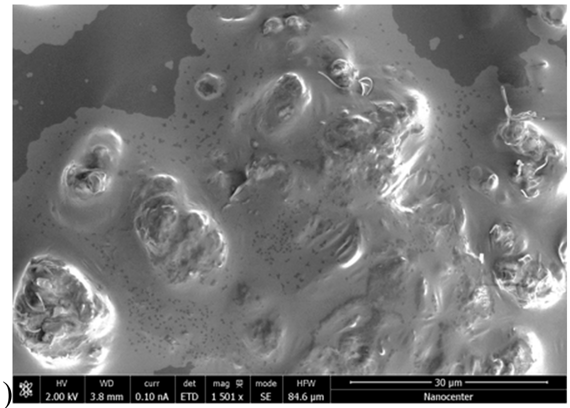
HV WD curr det mag mode HFW
2.00 kV 3.8 mm 0.10 nA ETD 1 501 x SE 84.6 µm
30 µm
Nanocenter
j)

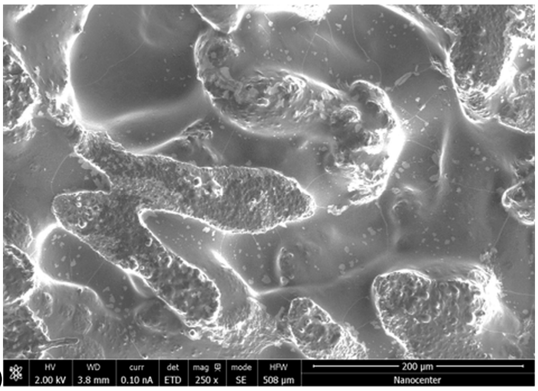
HV WD curr det mag mode HFW
2.00 kV 3.8 mm 0.10 nA ETD 250 x SE 508 µm
200 µm
Nanocenter
k)

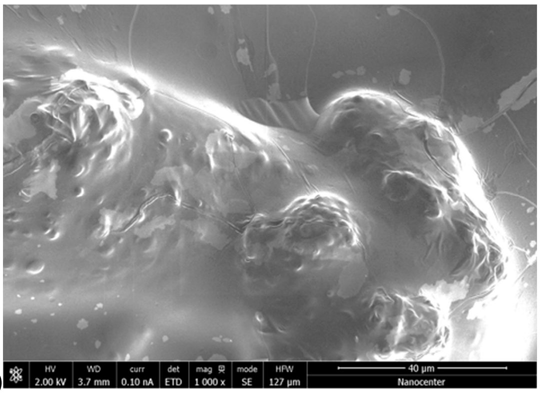
HV WD curr det mag mode HFW
2.00 kV 3.7 mm 0.10 nA ETD 1 000 x SE 127 µm
40 µm
Nanocenter
l)

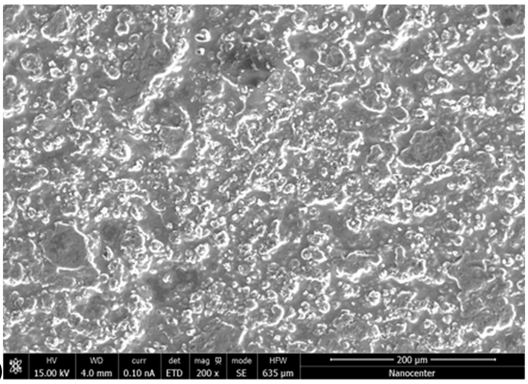
HV WD curr det mag mode HFW
15.00 kV 4.0 mm 0.10 nA ETD 200 x SE 635 µm
200 µm
Nanocenter
m)

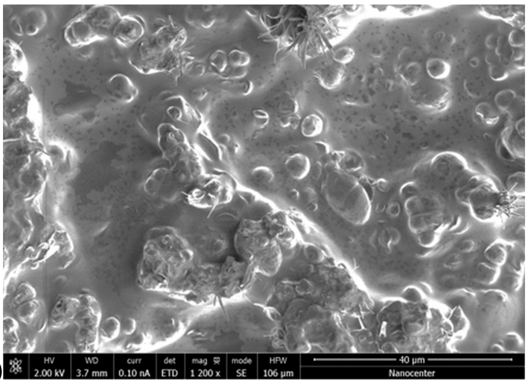
HV WD curr det mag mode HFW
2.00 kV 3.7 mm 0.10 nA ETD 1 200 x SE 106 µm
40 µm
Nanocenter
n)

Figure S3. SEM images of the films a) and b) PVA 0 (0 wt.% BW); c) and d) PVA 1 (3.5 wt.% BW); e) and f) PVA 2 (6.75 wt.% BW); g) and h) PVA 3 (6.75 wt.% BW); i) and j) PVA 4 (6.75 wt.% BW); k) and l) PVA 5 (6.75 wt.% BW) and m) and n) PVA 6 (6.75 wt.% BW).

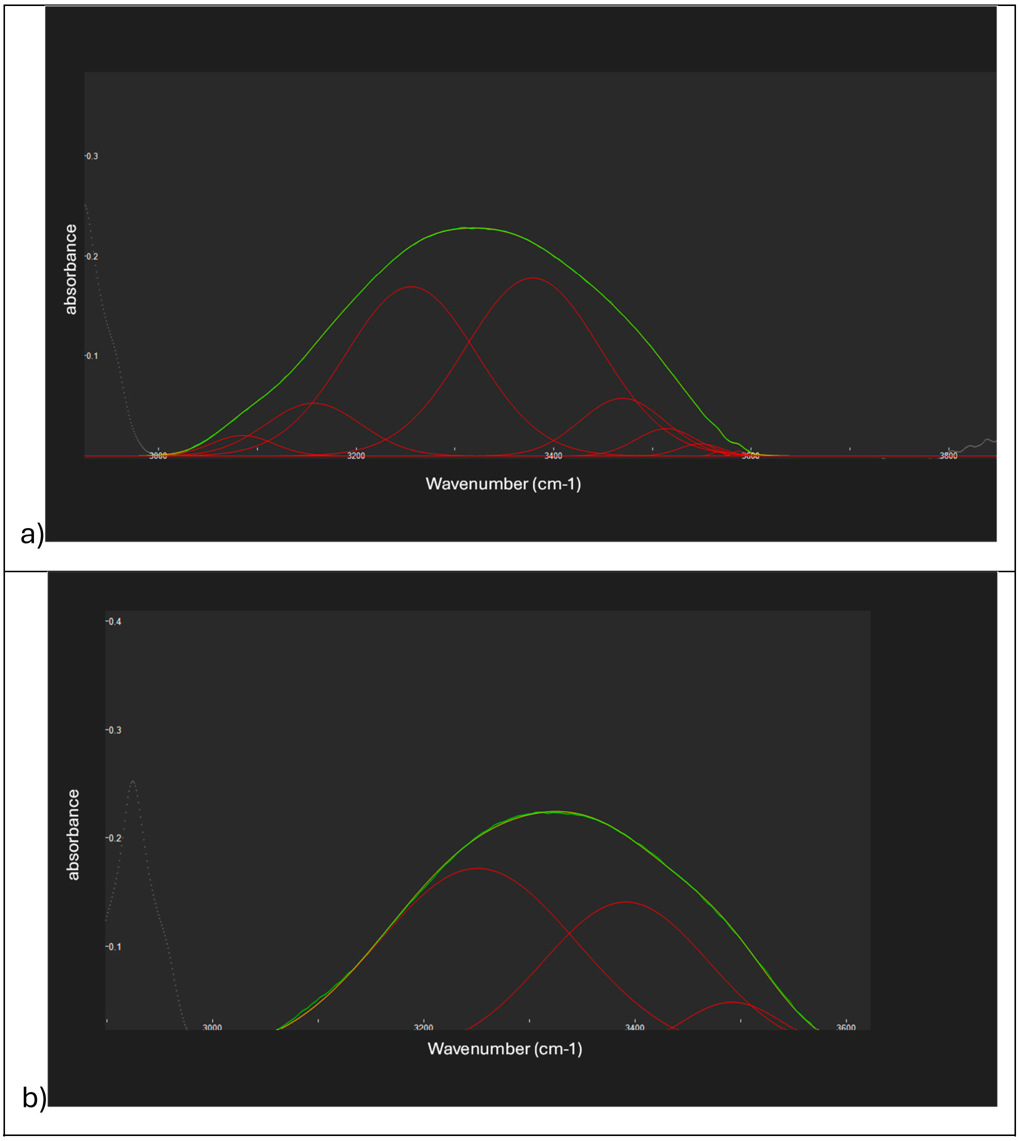

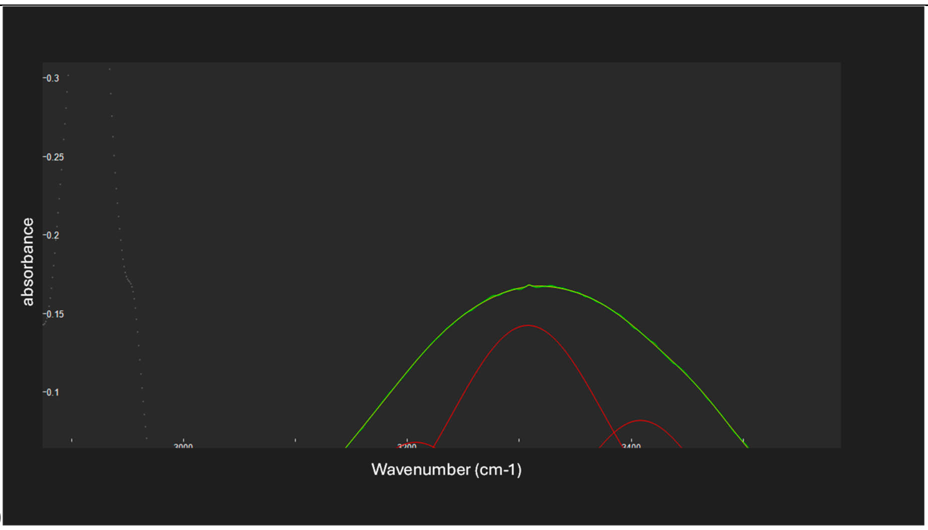

absorbance
0.3
0.25
0.2
0.15
0.1
Wavenumber (cm-1)

c)

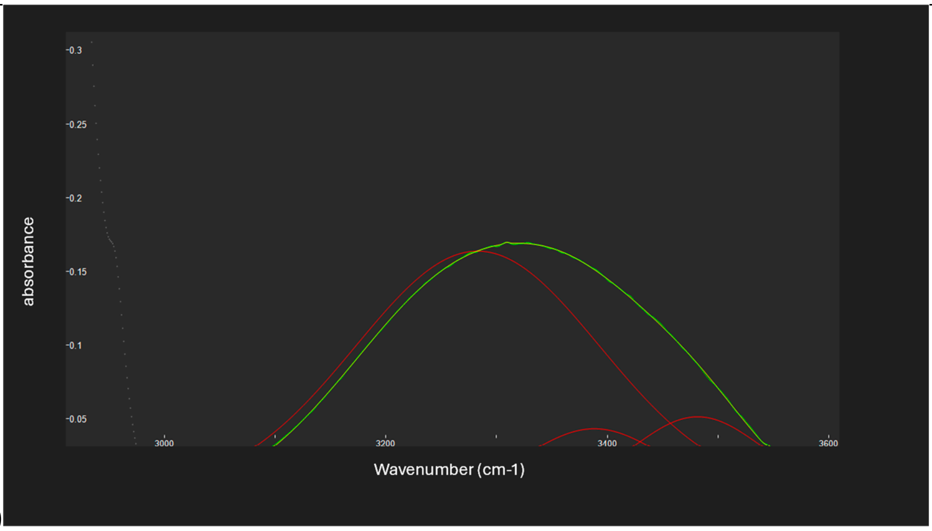

absorbance
0.3
0.25
0.2
0.15
0.1
0.05
3000
3200
3400
3600
Wavenumber (cm-1)

d)

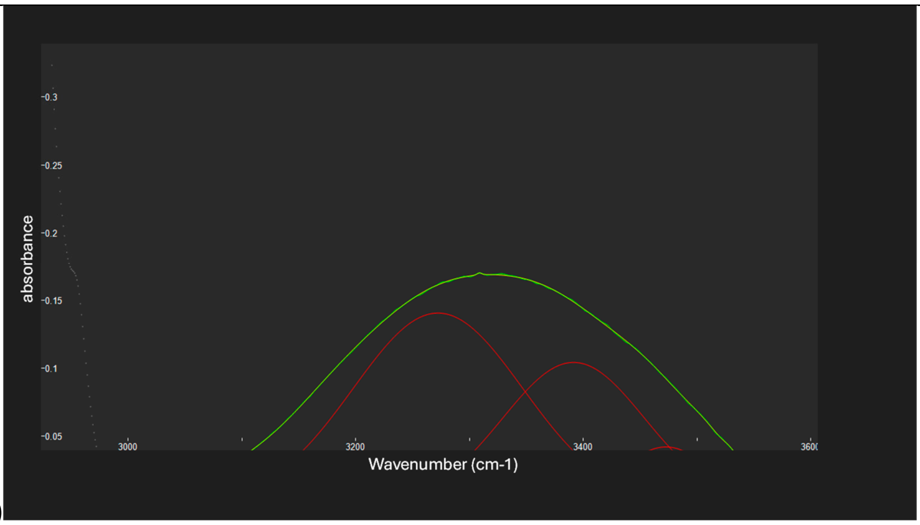

absorbance
0.3
0.25
0.2
0.15
0.1
0.05
3000
3200
3400
Wavenumber (cm-1)

e)

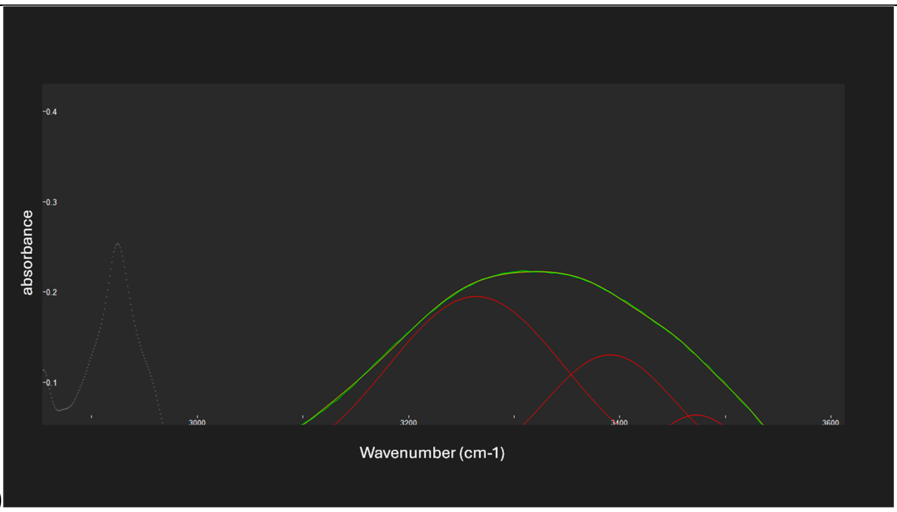

f)

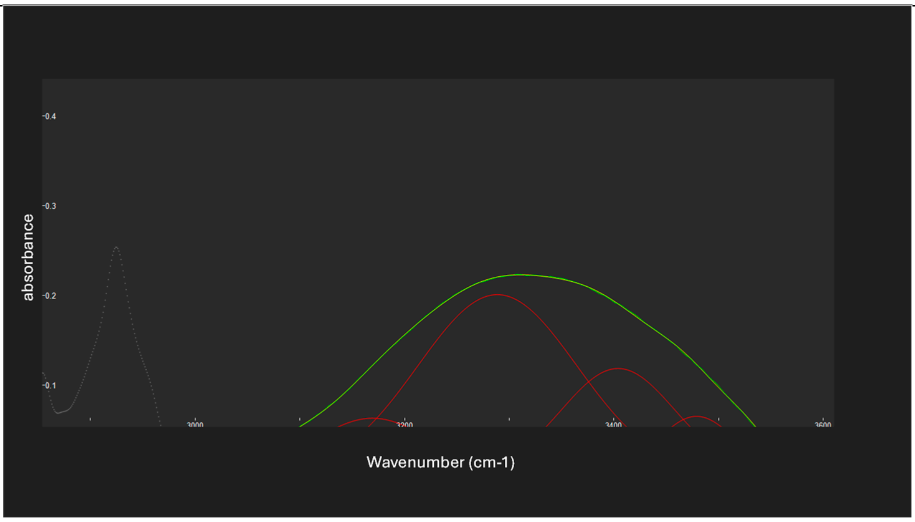

g)

Figure S4. Deconvoluted FTIR spectra in the region 3000-3600 cm^{-1} of PVA 0 (0 wt.% BW) a), PVA 1 (3.5 wt.% BW) b), PVA 2 (6.75 wt.% BW) c), PVA 3 (6.75 wt.% BW) d), PVA 4 (6.75 wt.% BW) e), PVA 5 (6.75 wt.% BW) f) and PVA 6 (6.75 wt.% BW) g)

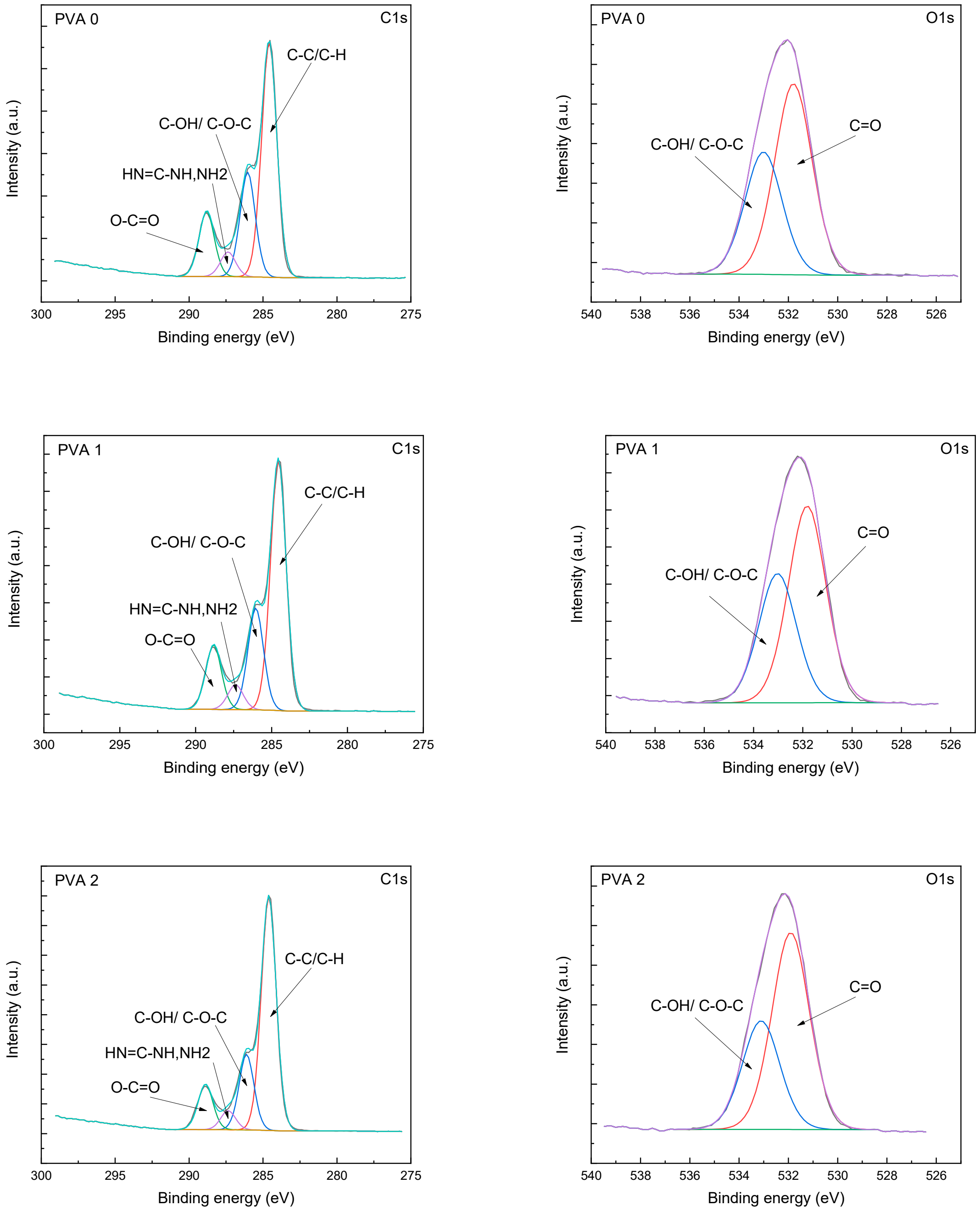

PVA 0
C1s
C-C/C-H
C-OH/ C-O-C
HN=C-NH,NH2
O-C=O
Intensity (a.u.)
300
295
290
285
280
275
Binding energy (eV)
PVA 0
O1s
C=O
C-OH/ C-O-C
Intensity (a.u.)
540
538
536
534
532
530
528
526
Binding energy (eV)
PVA 1
C1s
C-C/C-H
C-OH/ C-O-C
HN=C-NH,NH2
O-C=O
Intensity (a.u.)
300
295
290
285
280
275
Binding energy (eV)
PVA 1
O1s
C=O
C-OH/ C-O-C
Intensity (a.u.)
540
538
536
534
532
530
528
526
Binding energy (eV)
PVA 2
C1s
C-C/C-H
C-OH/ C-O-C
HN=C-NH,NH2
O-C=O
Intensity (a.u.)
300
295
290
285
280
275
Binding energy (eV)
PVA 2
O1s
C=O
C-OH/ C-O-C
Intensity (a.u.)
540
538
536
534
532
530
528
526
Binding energy (eV)

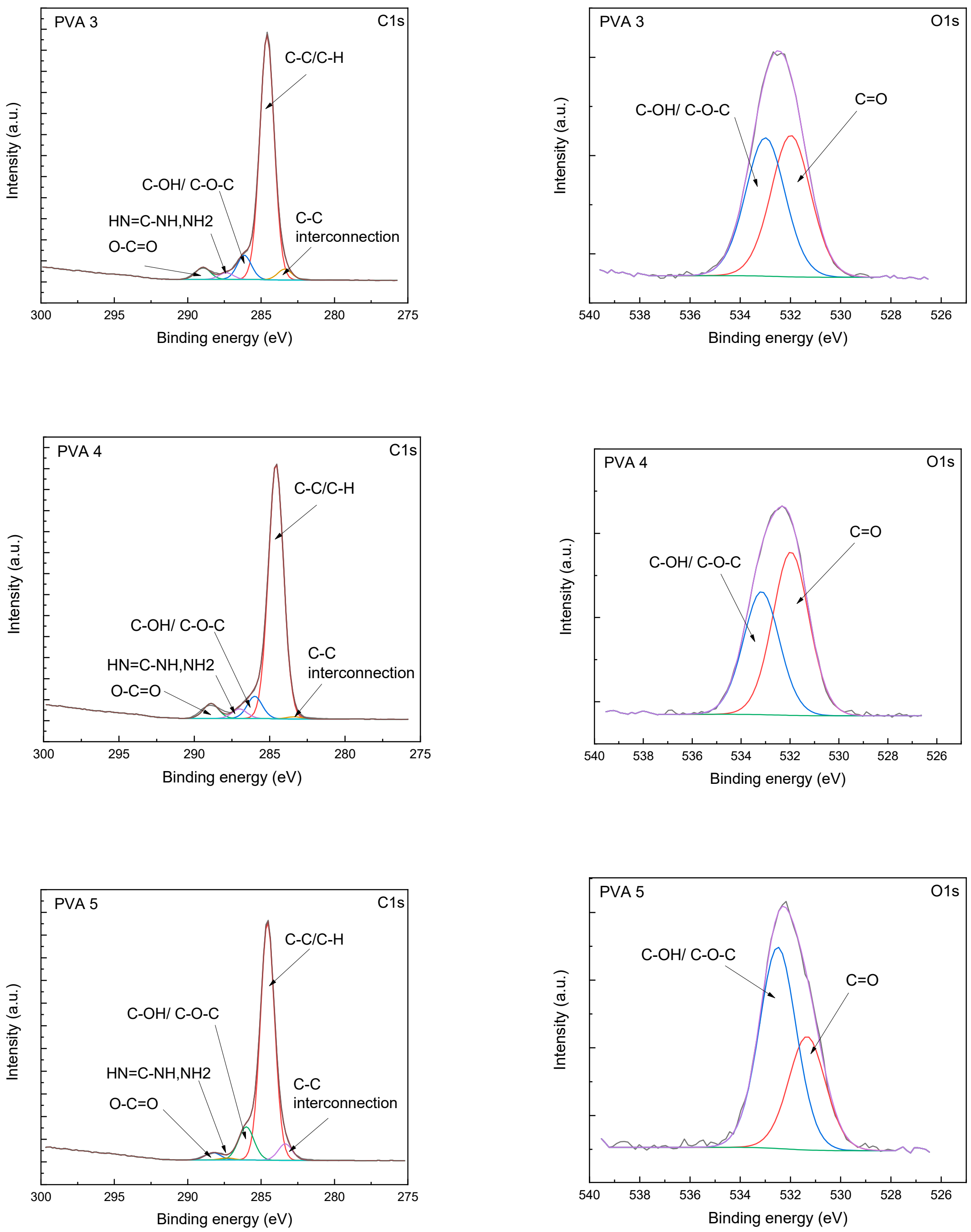
PVA 3
C1s
C-C/C-H
C-OH/ C-O-C
HN=C-NH,NH2
O-C=O
C-C
interconnection
Intensity (a.u.)
300 295 290 285 280 275
Binding energy (eV)
PVA 3
O1s
C-OH/ C-O-C
C=O
Intensity (a.u.)
540 538 536 534 532 530 528 526
Binding energy (eV)
PVA 4
C1s
C-C/C-H
C-OH/ C-O-C
HN=C-NH,NH2
O-C=O
C-C
interconnection
Intensity (a.u.)
300 295 290 285 280 275
Binding energy (eV)
PVA 4
O1s
C-OH/ C-O-C
C=O
Intensity (a.u.)
540 538 536 534 532 530 528 526
Binding energy (eV)
PVA 5
C1s
C-C/C-H
C-OH/ C-O-C
HN=C-NH,NH2
O-C=O
C-C
interconnection
Intensity (a.u.)
300 295 290 285 280 275
Binding energy (eV)
PVA 5
O1s
C-OH/ C-O-C
C=O
Intensity (a.u.)
540 538 536 534 532 530 528 526
Binding energy (eV)

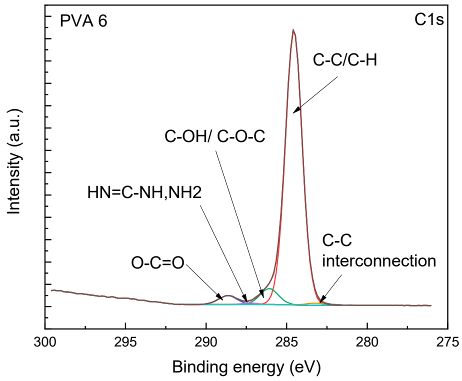

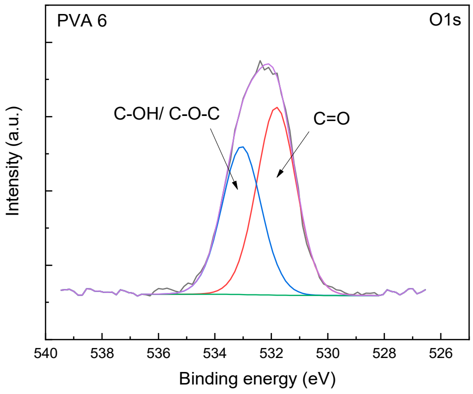

Figure S5. Deconvolution of C1s and O1s XPS spectra of PVA 0- PVA 6 films (PVA 0 (0 wt.% BW), PVA 1 (3.5 wt.% BW), PVA 2 (6.75 wt.% BW), PVA 3 (6.75 wt.% BW), PVA 4 (6.75 wt.% BW), PVA 5 (6.75 wt.% BW) and PVA 6 (6.75 wt.% BW))
